# Supplementary material for: Not So Similar: Different Ways of Nb(V) and Ta(V) Catecholate Complexation
Source: Molecules. 2023 Jun 22;28(13):4912. doi: 10.3390/molecules28134912 (PMC10361208; doi:10.3390/molecules28134912)

checkCIF/PLATON report

Structure factors have been supplied for datablock(s) gea203, gea279, gea354, gea355, gea361, gea381

THIS REPORT IS FOR GUIDANCE ONLY. IF USED AS PART OF A REVIEW PROCEDURE FOR PUBLICATION, IT SHOULD NOT REPLACE THE EXPERTISE OF AN EXPERIENCED CRYSTALLOGRAPHIC REFEREE.

No syntax errors found. CIF dictionary Interpreting this report

Datablock: gea203

Bond precision: C-C = 0.0150 A Wavelength=0.71073

Cell:	a=15.9081(7)	b=19.7180(8)	c=7.0292(3)
	alpha=90	beta=95.172(4)	gamma=90
Temperature:	130 K		

	Calculated	Reported
Volume	2195.91(16)	2195.91(16)
Space group	C c	C c
Hall group	C -2yc	C -2yc
Moiety formula	C18 H12 Nb O7, 2(O), 3(N)	?
Sum formula	C18 H12 N3 Nb O9	C18 H28 N3 Nb O9
Mr	507.22	523.34
Dx,g cm-3	1.534	1.583
Z	4	4
Mu (mm-1)	0.600	0.602
F000	1016.0	1080.0
F000'	1008.10	
h,k,lmax	19,23,8	19,23,8
Nref	4035[2023]	3181
Tmin,Tmax	0.965,0.970	0.961,1.000
Tmin'	0.835	

Correction method= # Reported T Limits: Tmin=0.961 Tmax=1.000
AbsCorr = MULTI-SCAN

Data completeness= 1.57/0.79 Theta(max)= 25.345

R(reflections)= 0.0436(2912) wR2(reflections)= 0.1057(3181)

S = 1.029 Npar= 280

The following ALERTS were generated. Each ALERT has the format
test-name_ALERT_alert-type_alert-level.
Click on the hyperlinks for more details of the test.

Alert level B

```
PLAT043_ALERT_1_B Calculated and Reported Mol. Weight Differ by ..      16.12 Check
```

Author Response: H-atoms were added directly to the formula

```
PLAT306_ALERT_2_B Isolated Oxygen Atom (H-atoms Missing ?) .......         O8 Check
```

Author Response: H-atoms of H2O and NH4 were not localized.

```
PLAT306_ALERT_2_B Isolated Oxygen Atom (H-atoms Missing ?) .......         O9 Check
```

Author Response: H-atoms of H2O and NH4 were not localized.

```
PLAT430_ALERT_2_B Short Inter D...A Contact  O1       ..N2       .       2.73 Ang.
                                              x,y,z  =     1_555 Check
PLAT430_ALERT_2_B Short Inter D...A Contact  O2       ..O8       .       2.80 Ang.
                                            x,y,1+z  =     1_556 Check
PLAT430_ALERT_2_B Short Inter D...A Contact  O3       ..O8       .       2.80 Ang.
                                        x,1-y,1/2+z  =     2_565 Check
PLAT430_ALERT_2_B Short Inter D...A Contact  O4       ..O9       .       2.76 Ang.
                                           x,y,-1+z  =     1_554 Check
PLAT430_ALERT_2_B Short Inter D...A Contact  O4       ..N3       .       2.84 Ang.
                                              x,y,z  =     1_555 Check
PLAT430_ALERT_2_B Short Inter D...A Contact  O5       ..N4       .       2.82 Ang.
                                              x,y,z  =     1_555 Check
PLAT430_ALERT_2_B Short Inter D...A Contact  O6       ..O9       .       2.72 Ang.
                                              x,y,z  =     1_555 Check
PLAT430_ALERT_2_B Short Inter D...A Contact  O7       ..O8       .       2.70 Ang.
                                              x,y,z  =     1_555 Check
PLAT430_ALERT_2_B Short Inter D...A Contact  O7       ..N4       .       2.82 Ang.
                                              x,y,z  =     1_555 Check
PLAT430_ALERT_2_B Short Inter D...A Contact  O9       ..N2       .       2.75 Ang.
                                  1/2+x,1/2-y,1/2+z  =     4_555 Check
```

Alert level C

```
PLAT041_ALERT_1_C Calc. and Reported SumFormula    Strings  Differ     Please Check
PLAT044_ALERT_1_C Calculated and Reported Density Dx  Differ by ..     0.0488 Check
PLAT048_ALERT_1_C MoietyFormula Not Given (or Incomplete) ........     Please Check
PLAT068_ALERT_1_C Reported F000 Differs from Calcd (or Missing)...     Please Check
PLAT090_ALERT_3_C Poor Data / Parameter Ratio (Zmax > 18) ........       7.13 Note
PLAT234_ALERT_4_C Large Hirshfeld Difference C17      --C18      .       0.16 Ang.
PLAT342_ALERT_3_C Low Bond Precision on  C-C Bonds ...............      0.015 Ang.
PLAT911_ALERT_3_C Missing FCF Refl Between Thmin & STh/L=    0.600         22 Report
PLAT975_ALERT_2_C Check Calcd Resid. Dens.  0.80Ang From N2      .       0.52 eA-3
PLAT975_ALERT_2_C Check Calcd Resid. Dens.  0.76Ang From N2      .       0.51 eA-3
PLAT975_ALERT_2_C Check Calcd Resid. Dens.  0.52Ang From N3      .       0.47 eA-3
```

```
PLAT975_ALERT_2_C Check Calcd Resid. Dens.  1.06Ang From N2      .       0.46 eA-3
PLAT975_ALERT_2_C Check Calcd Resid. Dens.  0.53Ang From N2      .       0.45 eA-3
PLAT975_ALERT_2_C Check Calcd Resid. Dens.  0.86Ang From N3      .       0.44 eA-3
PLAT975_ALERT_2_C Check Calcd Resid. Dens.  0.51Ang From N3      .       0.43 eA-3
PLAT975_ALERT_2_C Check Calcd Resid. Dens.  0.84Ang From N4      .       0.41 eA-3
PLAT975_ALERT_2_C Check Calcd Resid. Dens.  0.44Ang From N3      .       0.41 eA-3
PLAT975_ALERT_2_C Check Calcd Resid. Dens.  0.89Ang From N4      .       0.41 eA-3
```

Alert level G

```
FORMU01_ALERT_2_G  There is a discrepancy between the atom counts in the
            _chemical_formula_sum and the formula from the _atom_site* data.
            Atom count from _chemical_formula_sum:C18 H28 N3 Nb1 O9
            Atom count from the _atom_site data:  C18 H12 N3 Nb1 O9
CELLZ01_ALERT_1_G Difference between formula and atom_site contents detected.
CELLZ01_ALERT_1_G WARNING: H atoms missing from atom site list. Is this intentional?
           From the CIF: _cell_formula_units_Z    4
           From the CIF: _chemical_formula_sum  C18 H28 N3 Nb O9
           TEST: Compare cell contents of formula and atom_site data

           atom    Z*formula  cif sites diff
           C         72.00     72.00    0.00
           H        112.00     48.00   64.00
           N         12.00     12.00    0.00
           Nb         4.00      4.00    0.00
           O         36.00     36.00    0.00
PLAT794_ALERT_5_G Tentative Bond Valency for Nb1       (V)       .       4.92 Info
PLAT910_ALERT_3_G Missing # of FCF Reflection(s) Below Theta(Min).          4 Note
PLAT912_ALERT_4_G Missing # of FCF Reflections Above STh/L=  0.600          1 Note
PLAT915_ALERT_3_G No Flack x Check Done: Low Friedel Pair Coverage         59 %
PLAT933_ALERT_2_G Number of HKL-OMIT Records in Embedded .res File         53 Note
PLAT941_ALERT_3_G Average HKL Measurement Multiplicity ..........         2.7 Low
PLAT961_ALERT_5_G Dataset Contains no Negative Intensities .......     Please Check
PLAT978_ALERT_2_G Number C-C Bonds with Positive Residual Density.          0 Info
```

```
   0 ALERT level A = Most likely a serious problem - resolve or explain
  13 ALERT level B = A potentially serious problem, consider carefully
  18 ALERT level C = Check. Ensure it is not caused by an omission or oversight
  11 ALERT level G = General information/check it is not something unexpected

   7 ALERT type 1 CIF construction/syntax error, inconsistent or missing data
  25 ALERT type 2 Indicator that the structure model may be wrong or deficient
   6 ALERT type 3 Indicator that the structure quality may be low
   2 ALERT type 4 Improvement, methodology, query or suggestion
   2 ALERT type 5 Informative message, check
```

Datablock: gea381

Bond precision: C-C = 0.0022 A   Wavelength=0.71073

Cell:	a=8.7876(3)	b=14.0120(6)	c=15.4138(6)
	alpha=82.323(1)	beta=76.607(1)	gamma=76.500(1)
Temperature:	150 K		

	Calculated	Reported
Volume	1788.95(12)	1788.95(12)
Space group	P -1	P -1
Hall group	-P 1	-P 1
Moiety formula	C36 H31 K2 Nb O13, H2 O	?
Sum formula	C36 H33 K2 Nb O14	C36 H33 K2 Nb O14
Mr	860.73	860.73
Dx,g cm-3	1.598	1.598
Z	2	2
Mu (mm-1)	0.639	0.639
F000	880.0	880.0
F000’	877.03	
h,k,lmax	15,24,26	14,23,26
Nref	19671	16957
Tmin,Tmax	0.912,0.926	0.673,0.748
Tmin’	0.880	

Correction method= # Reported T Limits: Tmin=0.673 Tmax=0.748
AbsCorr = MULTI-SCAN

Data completeness= 0.862 Theta(max)= 38.139

R(reflections)= 0.0405(12331) wR2(reflections)= 0.0867(16957)

S = 1.017 Npar= 505

The following ALERTS were generated. Each ALERT has the format
test-name_ALERT_alert-type_alert-level.
Click on the hyperlinks for more details of the test.

Alert level B

PLAT420_ALERT_2_B D-H Bond Without Acceptor O1 --H13 . Please Check

Author Response: H-atoms were found directly from experiment and demonstrate contacts of other types.

PLAT420_ALERT_2_B D-H Bond Without Acceptor O12 --H31 . Please Check

Author Response: H-atoms were found directly from experiment and demonstrate contacts of other types.

Alert level C

```
PLAT048_ALERT_1_C MoietyFormula Not Given (or Incomplete) ........     Please Check
PLAT354_ALERT_3_C Short  O-H (X0.82,N0.98A)  O14       - H26      .       0.71 Ang.
PLAT906_ALERT_3_C Large K Value in the Analysis of Variance ......      3.069 Check
PLAT911_ALERT_3_C Missing FCF Refl Between Thmin & STh/L=    0.600         25 Report
PLAT977_ALERT_2_C Check Negative Difference Density on H3         .      -0.33 eA-3
PLAT977_ALERT_2_C Check Negative Difference Density on H36        .      -0.33 eA-3
```

Alert level G

```
PLAT004_ALERT_5_G Polymeric Structure Found with Maximum Dimension          1 Info
PLAT154_ALERT_1_G The s.u.'s on the Cell Angles are Equal ..(Note)      0.001 Degree
PLAT232_ALERT_2_G Hirshfeld Test Diff (M-X)  Nb1      --O2        .       5.6 s.u.
PLAT232_ALERT_2_G Hirshfeld Test Diff (M-X)  Nb1      --O3        .       5.8 s.u.
PLAT764_ALERT_4_G Overcomplete CIF Bond List Detected (Rep/Expd) .       1.20 Ratio
PLAT774_ALERT_1_G Check   X-Y Bond in CIF: Nb1      --K1          ..      4.11 Ang.
PLAT779_ALERT_4_G Suspect or Irrelevant (Bond) Angle(s) in CIF ...      42.21 Deg.
              O3   -C7   -K1      1_555   1_555   1_555 ......     #  261 Check
PLAT780_ALERT_1_G Coordinates do not Form a Properly Connected Set     Please Do !
PLAT794_ALERT_5_G Tentative Bond Valency for Nb1       (V)        .       4.81 Info
PLAT804_ALERT_5_G Number of ARU-Code Packing Problem(s) in PLATON           1 Info
PLAT910_ALERT_3_G Missing # of FCF Reflection(s) Below Theta(Min).          2 Note
PLAT912_ALERT_4_G Missing # of FCF Reflections Above STh/L=  0.600       2587 Note
PLAT913_ALERT_3_G Missing # of Very Strong Reflections in FCF ....          2 Note
PLAT933_ALERT_2_G Number of HKL-OMIT Records in Embedded .res File          8 Note
PLAT941_ALERT_3_G Average HKL Measurement Multiplicity ..........         1.8 Low
PLAT961_ALERT_5_G Dataset Contains no Negative Intensities .......     Please Check
PLAT978_ALERT_2_G Number C-C Bonds with Positive Residual Density.         13 Info
PLAT992_ALERT_5_G Repd & Actual _reflns_number_gt Values Differ by          4 Check
```

```
   0 ALERT level A = Most likely a serious problem - resolve or explain
   2 ALERT level B = A potentially serious problem, consider carefully
   6 ALERT level C = Check. Ensure it is not caused by an omission or oversight
  18 ALERT level G = General information/check it is not something unexpected

   4 ALERT type 1 CIF construction/syntax error, inconsistent or missing data
   8 ALERT type 2 Indicator that the structure model may be wrong or deficient
   6 ALERT type 3 Indicator that the structure quality may be low
   3 ALERT type 4 Improvement, methodology, query or suggestion
   5 ALERT type 5 Informative message, check
```

Datablock: gea354

```
Bond precision:    C-C = 0.0221 A            Wavelength=0.71073

Cell:               a=7.4397(5)       b=21.460(3)       c=16.1460(12)
                    alpha=90          beta=90           gamma=90
Temperature:        130 K
```

	Calculated	Reported
Volume	2577.8(4)	2577.9(5)
Space group	P n a 21	P n a 21
Hall group	P 2c -2n	P 2c -2n
Moiety formula	C18 H12 Cs3 Nb O10	?
Sum formula	C18 H12 Cs3 Nb O10	C18 H18 Cs3 Nb O10
Mr	879.92	885.96
Dx,g cm-3	2.267	2.283
Z	4	4
Mu (mm-1)	4.688	4.688
F000	1624.0	1648.0
F000'	1611.81	
h,k,lmax	10,29,22	10,26,21
Nref	7195[3718]	4446
Tmin,Tmax	0.338,0.495	0.979,1.000
Tmin'	0.298	

Correction method= # Reported T Limits: Tmin=0.979 Tmax=1.000
AbsCorr = MULTI-SCAN

Data completeness= 1.20/0.62 Theta(max)= 29.494

R(reflections)= 0.0513(3579) wR2(reflections)= 0.1089(4446)

S = 1.061 Npar= 289

The following ALERTS were generated. Each ALERT has the format
test-name_ALERT_alert-type_alert-level.
Click on the hyperlinks for more details of the test.

Alert level B

PLAT342_ALERT_3_B Low Bond Precision on C-C Bonds .... 0.02211 Ang.
PLAT601_ALERT_2_B Unit Cell Contains Solvent Accessible VOIDS of . 112 Ang**3

Author Response: Complex composition was found from analytical data

PLAT780_ALERT_1_B Coordinates do not Form a Properly Connected Set Please Do !

Alert level C

PLAT041_ALERT_1_C Calc. and Reported SumFormula Strings Differ Please Check
PLAT043_ALERT_1_C Calculated and Reported Mol. Weight Differ by .. 6.04 Check
PLAT048_ALERT_1_C MoietyFormula Not Given (or Incomplete) .... Please Check
PLAT068_ALERT_1_C Reported F000 Differs from Calcd (or Missing)... Please Check
PLAT220_ALERT_2_C NonSolvent Resd 1 C Ueq(max)/Ueq(min) Range 3.8 Ratio
PLAT220_ALERT_2_C NonSolvent Resd 1 O Ueq(max)/Ueq(min) Range 3.3 Ratio

```
PLAT234_ALERT_4_C Large Hirshfeld Difference C3       --C4       .       0.18 Ang.
PLAT234_ALERT_4_C Large Hirshfeld Difference C14      --C15      .       0.17 Ang.
PLAT234_ALERT_4_C Large Hirshfeld Difference C17      --C18      .       0.22 Ang.
PLAT241_ALERT_2_C High   'MainMol' Ueq as Compared to Neighbors of        C17 Check
PLAT906_ALERT_3_C Large K Value in the Analysis of Variance ......      2.251 Check
PLAT910_ALERT_3_C Missing # of FCF Reflection(s) Below Theta(Min).          7 Note
PLAT911_ALERT_3_C Missing FCF Refl Between Thmin & STh/L=    0.600         19 Report
PLAT971_ALERT_2_C Check Calcd Resid. Dens.  2.56Ang From O2W             1.51 eA-3
PLAT975_ALERT_2_C Check Calcd Resid. Dens.  1.06Ang From O1W     .       0.78 eA-3
```

Alert level G

```
FORMU01_ALERT_2_G  There is a discrepancy between the atom counts in the
            _chemical_formula_sum and the formula from the _atom_site* data.
            Atom count from _chemical_formula_sum:C18 H18 Cs3 Nb1 O10
            Atom count from the _atom_site data:  C18 H12 Cs3 Nb1 O10
CELLZ01_ALERT_1_G Difference between formula and atom_site contents detected.
CELLZ01_ALERT_1_G WARNING: H atoms missing from atom site list. Is this intentional?
           From the CIF: _cell_formula_units_Z    4
           From the CIF: _chemical_formula_sum  C18 H18 Cs3 Nb O10
           TEST: Compare cell contents of formula and atom_site data

           atom    Z*formula  cif sites diff
           C         72.00     72.00    0.00
           H         72.00     48.00   24.00
           Cs        12.00     12.00    0.00
           Nb         4.00      4.00    0.00
           O         40.00     40.00    0.00
PLAT003_ALERT_2_G Number of Uiso or Uij Restrained non-H Atoms ...          2 Report
PLAT004_ALERT_5_G Polymeric Structure Found with Maximum Dimension          3 Info
PLAT186_ALERT_4_G The CIF-Embedded .res File Contains ISOR Records          2 Report
PLAT343_ALERT_2_G Unusual          Angle Range in Main Residue for        C4 Check
PLAT764_ALERT_4_G Overcomplete CIF Bond List Detected (Rep/Expd) .       1.49 Ratio
PLAT779_ALERT_4_G Suspect or Irrelevant (Bond) Angle(s) in CIF ...      44.60 Deg.
              O2   -C6   -CS2     1_555   1_555   1_655 ......     #  338 Check
PLAT794_ALERT_5_G Tentative Bond Valency for Nb1       (V)       .       4.82 Info
PLAT850_ALERT_4_G Check Flack Parameter Exact Value 0.00 with s.u.       0.03 Check
PLAT860_ALERT_3_G Number of Least-Squares Restraints ............         13 Note
PLAT912_ALERT_4_G Missing # of FCF Reflections Above STh/L=  0.600        493 Note
PLAT915_ALERT_3_G No Flack x Check Done: Low Friedel Pair Coverage         38 %
PLAT933_ALERT_2_G Number of HKL-OMIT Records in Embedded .res File        101 Note
PLAT941_ALERT_3_G Average HKL Measurement Multiplicity ..........         2.7 Low
PLAT951_ALERT_5_G Calculated (ThMax) and CIF-Reported Kmax Differ           3 Units
PLAT957_ALERT_1_G Calculated (ThMax) and Actual (FCF) Kmax Differ           3 Units
PLAT961_ALERT_5_G Dataset Contains no Negative Intensities .......     Please Check
PLAT978_ALERT_2_G Number C-C Bonds with Positive Residual Density.          0 Info
```

---

0 **ALERT level A** = Most likely a serious problem - resolve or explain
3 **ALERT level B** = A potentially serious problem, consider carefully
15 **ALERT level C** = Check. Ensure it is not caused by an omission or oversight
20 **ALERT level G** = General information/check it is not something unexpected

8 ALERT type 1 CIF construction/syntax error, inconsistent or missing data

```
 11 ALERT type 2 Indicator that the structure model may be wrong or deficient
  7 ALERT type 3 Indicator that the structure quality may be low
  8 ALERT type 4 Improvement, methodology, query or suggestion
  4 ALERT type 5 Informative message, check
```

Datablock: gea355

Bond precision: C-C = 0.0155 A   Wavelength=0.71073

Cell:	a=11.4815(5)	b=11.8561(4)	c=32.1364(9)
	alpha=90	beta=99.974(1)	gamma=90
Temperature:	296 K		

	Calculated	Reported
Volume	4308.5(3)	4308.5(3)
Space group	P 21/c	P 21/c
Hall group	-P 2ybc	-P 2ybc
Moiety formula	2(C36 H24 O13 Ta2), N2, 0.25(O2), 4.2(O), 6(N)	?
Sum formula	C72 H48 N8 O30.70 Ta4	C36 H24 N4 O15.35 Ta2
Mr	2240.19	1120.09
Dx,g cm-3	1.727	1.727
Z	2	4
Mu (mm-1)	5.144	5.144
F000	2147.2	2147.0
F000’	2142.82	
h,k,lmax	16,17,46	16,16,46
Nref	13679	11857
Tmin,Tmax	0.583,0.687	0.642,0.746
Tmin’	0.276	

Correction method= # Reported T Limits: Tmin=0.642 Tmax=0.746
AbsCorr = MULTI-SCAN

Data completeness= 0.867   Theta(max)= 30.952

R(reflections)= 0.0651(7085)   wR2(reflections)= 0.1780(11857)

S = 1.037   Npar= 485

```
The following ALERTS were generated. Each ALERT has the format
       test-name_ALERT_alert-type_alert-level.
Click on the hyperlinks for more details of the test.
```

Alert level A

```
PLAT201_ALERT_2_A Isotropic non-H Atoms in Main Residue(s) .......         14 Report
              O3A       O4B       C7B       C8B       C9B                 etc.
```

Author Response: Disordering of catechol ligands with small occupancies

```
PLAT430_ALERT_2_A Short Inter D...A Contact  O4B      ..O16B     .       2.37 Ang.
                                         1-x,1/2+y,1/2-z  =      2_655 Check
```

Author Response: SOF(O4B) = 0.6, SOF(O16B) = 0.4

```
PLAT430_ALERT_2_A Short Inter D...A Contact  O8B      ..N1       .       2.54 Ang.
                                                   x,y,z  =      1_555 Check
```

Author Response: SOF(O4B) = 0.6, SOF(O16B) = 0.4

Alert level B

```
PLAT430_ALERT_2_B Short Inter D...A Contact  O1       ..N4       .       2.72 Ang.
                                                   x,y,z  =      1_555 Check
```

Author Response: SOF(O4B) = 0.6, SOF(O16B) = 0.4

```
PLAT430_ALERT_2_B Short Inter D...A Contact  O4B      ..O16A     .       2.80 Ang.
                                         1-x,1/2+y,1/2-z  =      2_655 Check
```

Author Response: SOF(O4B) = 0.6, SOF(O16B) = 0.4

```
PLAT430_ALERT_2_B Short Inter D...A Contact  O5       ..O16A     .       2.84 Ang.
                                                   x,y,z  =      1_555 Check
```

Author Response: SOF(O4B) = 0.6, SOF(O16B) = 0.4

```
PLAT430_ALERT_2_B Short Inter D...A Contact  O7B      ..O14      .       2.73 Ang.
                                                 1+x,y,z  =      1_655 Check
```

Author Response: SOF(O4B) = 0.6, SOF(O16B) = 0.4

```
PLAT430_ALERT_2_B Short Inter D...A Contact  O8A      ..N1       .       2.81 Ang.
                                                       x,y,z  =      1_555 Check
```

Author Response: SOF(O4B) = 0.6, SOF(O16B) = 0.4

```
PLAT430_ALERT_2_B Short Inter D...A Contact  O8B      ..N2       .       2.80 Ang.
                                                       x,y,z  =      1_555 Check
```

Author Response: SOF(O4B) = 0.6, SOF(O16B) = 0.4

```
PLAT430_ALERT_2_B Short Inter D...A Contact  O10      ..O16B     .       2.71 Ang.
                                                       x,y,z  =      1_555 Check
```

Author Response: SOF(O4B) = 0.6, SOF(O16B) = 0.4

```
PLAT430_ALERT_2_B Short Inter D...A Contact  O10      ..O16A     .       2.79 Ang.
                                                       x,y,z  =      1_555 Check
```

Author Response: SOF(O4B) = 0.6, SOF(O16B) = 0.4

```
PLAT430_ALERT_2_B Short Inter D...A Contact  O11      ..N2       .       2.75 Ang.
                                                 1-x,1-y,1-z  =      3_666 Check
```

Author Response: SOF(O4B) = 0.6, SOF(O16B) = 0.4

```
PLAT430_ALERT_2_B Short Inter D...A Contact  O11      ..N1       .       2.77 Ang.
                                                 1-x,1-y,1-z  =      3_666 Check
```

Author Response: SOF(O4B) = 0.6, SOF(O16B) = 0.4

```
PLAT430_ALERT_2_B Short Inter D...A Contact  O12A     ..N5B      .       2.82 Ang.
                                                       x,y,z  =      1_555 Check
```

Author Response: SOF(O4B) = 0.6, SOF(O16B) = 0.4

```
PLAT430_ALERT_2_B Short Inter D...A Contact  O12B     ..N5B      .       2.59 Ang.
                                                       x,y,z  =      1_555 Check
```

Author Response: SOF(O4B) = 0.6, SOF(O16B) = 0.4

```
PLAT430_ALERT_2_B Short Inter D...A Contact  O12B     ..N5A      .       2.78 Ang.
                                                       x,y,z  =      1_555 Check
```

Author Response: SOF(O4B) = 0.6, SOF(O16B) = 0.4

```
PLAT430_ALERT_2_B Short Inter D...A Contact  O13A     ..N2       .      2.82 Ang.
                                                    x,y,z  =     1_555 Check
```

Author Response: SOF(O4B) = 0.6, SOF(O16B) = 0.4

```
PLAT430_ALERT_2_B Short Inter D...A Contact  O13B     ..N2       .      2.64 Ang.
                                                    x,y,z  =     1_555 Check
```

Author Response: SOF(O4B) = 0.6, SOF(O16B) = 0.4

```
PLAT430_ALERT_2_B Short Inter D...A Contact  N4       ..O3B      .      2.83 Ang.
                                                    x,y,z  =     1_555 Check
```

Author Response: SOF(O4B) = 0.6, SOF(O16B) = 0.4

```
PLAT430_ALERT_2_B Short Inter D...A Contact  O3B      ..O8       .      2.68 Ang.
                                         1-x,-1/2+y,1/2-z  =     2_645 Check
```

Author Response: SOF(O4B) = 0.6, SOF(O16B) = 0.4

```
PLAT430_ALERT_2_B Short Inter D...A Contact  O4A      ..O16B     .      2.68 Ang.
                                          1-x,1/2+y,1/2-z  =     2_655 Check
```

Author Response: SOF(O4B) = 0.6, SOF(O16B) = 0.4

```
PLAT975_ALERT_2_B Check Calcd Resid. Dens.  1.02Ang From O14     .      1.63 eA-3
```

Alert level C

```
PLAT048_ALERT_1_C MoietyFormula Not Given (or Incomplete) ........    Please Check
PLAT202_ALERT_3_C Isotropic non-H Atoms in Anion/Solvent .........         3 Check
              O14     O16A    N5A
PLAT220_ALERT_2_C NonSolvent   Resd 1  C   Ueq(max)/Ueq(min) Range       5.4 Ratio
PLAT234_ALERT_4_C Large Hirshfeld Difference C34      --C35      .      0.19 Ang.
PLAT241_ALERT_2_C High   'MainMol' Ueq as Compared to Neighbors of        O6 Check
PLAT241_ALERT_2_C High   'MainMol' Ueq as Compared to Neighbors of       O10 Check
PLAT241_ALERT_2_C High   'MainMol' Ueq as Compared to Neighbors of       O11 Check
PLAT241_ALERT_2_C High   'MainMol' Ueq as Compared to Neighbors of       C34 Check
PLAT242_ALERT_2_C Low    'MainMol' Ueq as Compared to Neighbors of       C32 Check
PLAT342_ALERT_3_C Low Bond Precision on  C-C Bonds ..............    0.0155 Ang.
PLAT411_ALERT_2_C Short Inter H...H Contact  H34      ..H34      .      2.08 Ang.
                                                 2-x,-y,1-z  =     3_756 Check
PLAT430_ALERT_2_C Short Inter D...A Contact  O14      ..N4       .      2.88 Ang.
                                                    x,y,z  =     1_555 Check
```

Author Response: SOF(O4B) = 0.6, SOF(O16B) = 0.4

```
PLAT906_ALERT_3_C Large K Value in the Analysis of Variance ......     13.915 Check
PLAT906_ALERT_3_C Large K Value in the Analysis of Variance ......      2.577 Check
PLAT911_ALERT_3_C Missing FCF Refl Between Thmin & STh/L=    0.600         20 Report
PLAT971_ALERT_2_C Check Calcd Resid. Dens.  1.02Ang From O14             1.63 eA-3
PLAT972_ALERT_2_C Check Calcd Resid. Dens.  1.11Ang From C24B           -1.94 eA-3
PLAT975_ALERT_2_C Check Calcd Resid. Dens.  0.58Ang From O20     .       1.24 eA-3
PLAT975_ALERT_2_C Check Calcd Resid. Dens.  0.77Ang From O20     .       1.01 eA-3
PLAT975_ALERT_2_C Check Calcd Resid. Dens.  0.61Ang From N3      .       0.79 eA-3
PLAT975_ALERT_2_C Check Calcd Resid. Dens.  0.45Ang From O14     .       0.77 eA-3
PLAT975_ALERT_2_C Check Calcd Resid. Dens.  0.76Ang From O20     .       0.76 eA-3
PLAT977_ALERT_2_C Check Negative Difference Density on H23A      .      -0.60 eA-3
PLAT977_ALERT_2_C Check Negative Difference Density on H12B      .      -0.35 eA-3
```

Alert level G

```
PLAT045_ALERT_1_G Calculated and Reported Z Differ by a Factor ...      0.500 Check
PLAT068_ALERT_1_G Reported F000 Differs from Calcd (or Missing)...     Please Check
PLAT300_ALERT_4_G Atom Site Occupancy of Ta2A       Constrained at        0.5 Check
PLAT300_ALERT_4_G Atom Site Occupancy of Ta2B       Constrained at        0.5 Check
PLAT300_ALERT_4_G Atom Site Occupancy of O3A        Constrained at        0.6 Check
PLAT300_ALERT_4_G Atom Site Occupancy of O4B        Constrained at        0.6 Check
PLAT300_ALERT_4_G Atom Site Occupancy of O7A        Constrained at        0.5 Check
PLAT300_ALERT_4_G Atom Site Occupancy of O7B        Constrained at        0.5 Check
PLAT300_ALERT_4_G Atom Site Occupancy of O8A        Constrained at        0.5 Check
PLAT300_ALERT_4_G Atom Site Occupancy of O8B        Constrained at        0.5 Check
PLAT300_ALERT_4_G Atom Site Occupancy of O12A       Constrained at        0.5 Check
PLAT300_ALERT_4_G Atom Site Occupancy of O12B       Constrained at        0.5 Check
PLAT300_ALERT_4_G Atom Site Occupancy of O13A       Constrained at        0.5 Check
PLAT300_ALERT_4_G Atom Site Occupancy of O13B       Constrained at        0.5 Check
PLAT300_ALERT_4_G Atom Site Occupancy of O3B        Constrained at        0.4 Check
PLAT300_ALERT_4_G Atom Site Occupancy of O4A        Constrained at        0.4 Check
PLAT300_ALERT_4_G Atom Site Occupancy of C7B        Constrained at        0.6 Check
PLAT300_ALERT_4_G Atom Site Occupancy of C8B        Constrained at        0.6 Check
PLAT300_ALERT_4_G Atom Site Occupancy of C9B        Constrained at        0.6 Check
PLAT300_ALERT_4_G Atom Site Occupancy of C10B       Constrained at        0.6 Check
PLAT300_ALERT_4_G Atom Site Occupancy of C11        Constrained at        0.6 Check
PLAT300_ALERT_4_G Atom Site Occupancy of C12A       Constrained at        0.6 Check
PLAT300_ALERT_4_G Atom Site Occupancy of C13B       Constrained at        0.6 Check
PLAT300_ALERT_4_G Atom Site Occupancy of C14B       Constrained at        0.6 Check
PLAT300_ALERT_4_G Atom Site Occupancy of C15B       Constrained at        0.6 Check
PLAT300_ALERT_4_G Atom Site Occupancy of C16B       Constrained at        0.6 Check
PLAT300_ALERT_4_G Atom Site Occupancy of C17A       Constrained at        0.6 Check
PLAT300_ALERT_4_G Atom Site Occupancy of C18B       Constrained at        0.6 Check
PLAT300_ALERT_4_G Atom Site Occupancy of C19A       Constrained at        0.5 Check
PLAT300_ALERT_4_G Atom Site Occupancy of C19B       Constrained at        0.5 Check
PLAT300_ALERT_4_G Atom Site Occupancy of C20A       Constrained at        0.5 Check
PLAT300_ALERT_4_G Atom Site Occupancy of C20B       Constrained at        0.5 Check
PLAT300_ALERT_4_G Atom Site Occupancy of C21A       Constrained at        0.5 Check
PLAT300_ALERT_4_G Atom Site Occupancy of C21B       Constrained at        0.5 Check
PLAT300_ALERT_4_G Atom Site Occupancy of C22A       Constrained at        0.5 Check
PLAT300_ALERT_4_G Atom Site Occupancy of C22B       Constrained at        0.5 Check
PLAT300_ALERT_4_G Atom Site Occupancy of C23A       Constrained at        0.5 Check
PLAT300_ALERT_4_G Atom Site Occupancy of C23B       Constrained at        0.5 Check
PLAT300_ALERT_4_G Atom Site Occupancy of C24A       Constrained at        0.5 Check
PLAT300_ALERT_4_G Atom Site Occupancy of C24B       Constrained at        0.5 Check
PLAT300_ALERT_4_G Atom Site Occupancy of C25A       Constrained at        0.5 Check
PLAT300_ALERT_4_G Atom Site Occupancy of C25B       Constrained at        0.5 Check
```

```
PLAT300_ALERT_4_G Atom Site Occupancy of C25C       Constrained at        0.5 Check
PLAT300_ALERT_4_G Atom Site Occupancy of C26B       Constrained at        0.5 Check
PLAT300_ALERT_4_G Atom Site Occupancy of C27A       Constrained at        0.5 Check
PLAT300_ALERT_4_G Atom Site Occupancy of C28A       Constrained at        0.5 Check
PLAT300_ALERT_4_G Atom Site Occupancy of C28C       Constrained at        0.5 Check
PLAT300_ALERT_4_G Atom Site Occupancy of C28Y       Constrained at        0.5 Check
PLAT300_ALERT_4_G Atom Site Occupancy of C29B       Constrained at        0.5 Check
PLAT300_ALERT_4_G Atom Site Occupancy of C29X       Constrained at        0.5 Check
PLAT300_ALERT_4_G Atom Site Occupancy of C30A       Constrained at        0.5 Check
PLAT300_ALERT_4_G Atom Site Occupancy of C30B       Constrained at        0.5 Check
PLAT300_ALERT_4_G Atom Site Occupancy of C7A        Constrained at        0.4 Check
PLAT300_ALERT_4_G Atom Site Occupancy of C8A        Constrained at        0.4 Check
PLAT300_ALERT_4_G Atom Site Occupancy of C9A        Constrained at        0.4 Check
PLAT300_ALERT_4_G Atom Site Occupancy of C10A       Constrained at        0.4 Check
PLAT300_ALERT_4_G Atom Site Occupancy of C11B       Constrained at        0.4 Check
PLAT300_ALERT_4_G Atom Site Occupancy of C12B       Constrained at        0.4 Check
PLAT300_ALERT_4_G Atom Site Occupancy of C13A       Constrained at        0.4 Check
PLAT300_ALERT_4_G Atom Site Occupancy of C14A       Constrained at        0.4 Check
PLAT300_ALERT_4_G Atom Site Occupancy of C15A       Constrained at        0.4 Check
PLAT300_ALERT_4_G Atom Site Occupancy of C16A       Constrained at        0.4 Check
PLAT300_ALERT_4_G Atom Site Occupancy of C17B       Constrained at        0.4 Check
PLAT300_ALERT_4_G Atom Site Occupancy of C18A       Constrained at        0.4 Check
PLAT300_ALERT_4_G Atom Site Occupancy of H9B        Constrained at        0.6 Check
PLAT300_ALERT_4_G Atom Site Occupancy of H10B       Constrained at        0.6 Check
PLAT300_ALERT_4_G Atom Site Occupancy of H11        Constrained at        0.6 Check
PLAT300_ALERT_4_G Atom Site Occupancy of H12A       Constrained at        0.6 Check
PLAT300_ALERT_4_G Atom Site Occupancy of H15B       Constrained at        0.6 Check
PLAT300_ALERT_4_G Atom Site Occupancy of H16B       Constrained at        0.6 Check
PLAT300_ALERT_4_G Atom Site Occupancy of H17A       Constrained at        0.6 Check
PLAT300_ALERT_4_G Atom Site Occupancy of H18B       Constrained at        0.6 Check
PLAT300_ALERT_4_G Atom Site Occupancy of H20A       Constrained at        0.5 Check
PLAT300_ALERT_4_G Atom Site Occupancy of H20B       Constrained at        0.5 Check
PLAT300_ALERT_4_G Atom Site Occupancy of H21A       Constrained at        0.5 Check
PLAT300_ALERT_4_G Atom Site Occupancy of H21B       Constrained at        0.5 Check
PLAT300_ALERT_4_G Atom Site Occupancy of H22A       Constrained at        0.5 Check
PLAT300_ALERT_4_G Atom Site Occupancy of H22B       Constrained at        0.5 Check
PLAT300_ALERT_4_G Atom Site Occupancy of H23A       Constrained at        0.5 Check
PLAT300_ALERT_4_G Atom Site Occupancy of H23B       Constrained at        0.5 Check
PLAT300_ALERT_4_G Atom Site Occupancy of H27A       Constrained at        0.5 Check
PLAT300_ALERT_4_G Atom Site Occupancy of H28A       Constrained at        0.5 Check
PLAT300_ALERT_4_G Atom Site Occupancy of H28C       Constrained at        0.5 Check
PLAT300_ALERT_4_G Atom Site Occupancy of H28Y       Constrained at        0.5 Check
PLAT300_ALERT_4_G Atom Site Occupancy of H29B       Constrained at        0.5 Check
PLAT300_ALERT_4_G Atom Site Occupancy of H29X       Constrained at        0.5 Check
PLAT300_ALERT_4_G Atom Site Occupancy of H30A       Constrained at        0.5 Check
PLAT300_ALERT_4_G Atom Site Occupancy of H30B       Constrained at        0.5 Check
PLAT300_ALERT_4_G Atom Site Occupancy of H9A        Constrained at        0.4 Check
PLAT300_ALERT_4_G Atom Site Occupancy of H10A       Constrained at        0.4 Check
PLAT300_ALERT_4_G Atom Site Occupancy of H11B       Constrained at        0.4 Check
PLAT300_ALERT_4_G Atom Site Occupancy of H12B       Constrained at        0.4 Check
PLAT300_ALERT_4_G Atom Site Occupancy of H15A       Constrained at        0.4 Check
PLAT300_ALERT_4_G Atom Site Occupancy of H16A       Constrained at        0.4 Check
PLAT300_ALERT_4_G Atom Site Occupancy of H17B       Constrained at        0.4 Check
PLAT300_ALERT_4_G Atom Site Occupancy of H18A       Constrained at        0.4 Check
PLAT300_ALERT_4_G Atom Site Occupancy of N1         Constrained at        0.5 Check
PLAT300_ALERT_4_G Atom Site Occupancy of N2         Constrained at        0.5 Check
PLAT300_ALERT_4_G Atom Site Occupancy of O20        Constrained at       0.25 Check
```

```
PLAT300_ALERT_4_G Atom Site Occupancy of O14        Constrained at        0.6 Check
PLAT300_ALERT_4_G Atom Site Occupancy of O16A       Constrained at        0.6 Check
PLAT300_ALERT_4_G Atom Site Occupancy of N5A        Constrained at        0.6 Check
PLAT300_ALERT_4_G Atom Site Occupancy of O7         Constrained at       0.25 Check
PLAT300_ALERT_4_G Atom Site Occupancy of O8         Constrained at       0.25 Check
PLAT300_ALERT_4_G Atom Site Occupancy of O16B       Constrained at        0.4 Check
PLAT300_ALERT_4_G Atom Site Occupancy of N5B        Constrained at        0.4 Check
PLAT301_ALERT_3_G Main Residue  Disorder .............(Resd  1  )        61% Note
PLAT302_ALERT_4_G Anion/Solvent/Minor-Residue Disorder (Resd  2  )       100% Note
PLAT302_ALERT_4_G Anion/Solvent/Minor-Residue Disorder (Resd  3  )       100% Note
PLAT302_ALERT_4_G Anion/Solvent/Minor-Residue Disorder (Resd  4  )       100% Note
PLAT302_ALERT_4_G Anion/Solvent/Minor-Residue Disorder (Resd  5  )       100% Note
PLAT302_ALERT_4_G Anion/Solvent/Minor-Residue Disorder (Resd  8  )       100% Note
PLAT302_ALERT_4_G Anion/Solvent/Minor-Residue Disorder (Resd  9  )       100% Note
PLAT302_ALERT_4_G Anion/Solvent/Minor-Residue Disorder (Resd 10  )       100% Note
PLAT302_ALERT_4_G Anion/Solvent/Minor-Residue Disorder (Resd 11  )       100% Note
PLAT302_ALERT_4_G Anion/Solvent/Minor-Residue Disorder (Resd 12  )       100% Note
PLAT304_ALERT_4_G Non-Integer Number of Atoms in ..... (Resd  3  )       0.50 Check
PLAT304_ALERT_4_G Non-Integer Number of Atoms in ..... (Resd  4  )       0.60 Check
PLAT304_ALERT_4_G Non-Integer Number of Atoms in ..... (Resd  5  )       0.60 Check
PLAT304_ALERT_4_G Non-Integer Number of Atoms in ..... (Resd  8  )       0.60 Check
PLAT304_ALERT_4_G Non-Integer Number of Atoms in ..... (Resd  9  )       0.25 Check
PLAT304_ALERT_4_G Non-Integer Number of Atoms in ..... (Resd 10  )       0.25 Check
PLAT304_ALERT_4_G Non-Integer Number of Atoms in ..... (Resd 11  )       0.40 Check
PLAT304_ALERT_4_G Non-Integer Number of Atoms in ..... (Resd 12  )       0.40 Check
PLAT311_ALERT_2_G Isolated Disordered Oxygen Atom (No H's ?) .....        O14 Check
PLAT311_ALERT_2_G Isolated Disordered Oxygen Atom (No H's ?) .....       O16A Check
PLAT311_ALERT_2_G Isolated Disordered Oxygen Atom (No H's ?) .....         O7 Check
PLAT311_ALERT_2_G Isolated Disordered Oxygen Atom (No H's ?) .....         O8 Check
PLAT311_ALERT_2_G Isolated Disordered Oxygen Atom (No H's ?) .....       O16B Check
PLAT333_ALERT_2_G Large Aver C6-Ring C-C Dist C25A     -C27A     .       1.45 Ang.
PLAT335_ALERT_2_G Check Large C6 Ring C-C Range C7B      -C12A           0.26 Ang.
PLAT335_ALERT_2_G Check Large C6 Ring C-C Range C19B     -C24B           0.29 Ang.
PLAT335_ALERT_2_G Check Large C6 Ring C-C Range C25A     -C27A           0.28 Ang.
PLAT335_ALERT_2_G Check Large C6 Ring C-C Range C31      -C36            0.16 Ang.
PLAT335_ALERT_2_G Check Large C6 Ring C-C Range C13A     -C18A           0.22 Ang.
PLAT432_ALERT_2_G Short Inter X...Y Contact  O14      ..C17B     .       2.64 Ang.
                                                   -1+x,y,z  =     1_455 Check
PLAT432_ALERT_2_G Short Inter X...Y Contact  O14      ..C16A     .       2.89 Ang.
                                                   -1+x,y,z  =     1_455 Check
PLAT432_ALERT_2_G Short Inter X...Y Contact  O14      ..C18A     .       2.96 Ang.
                                                   -1+x,y,z  =     1_455 Check
PLAT432_ALERT_2_G Short Inter X...Y Contact  O8       ..C17A     .       1.95 Ang.
                                                      x,y,z  =     1_555 Check
PLAT432_ALERT_2_G Short Inter X...Y Contact  O8       ..C18B     .       2.22 Ang.
                                                      x,y,z  =     1_555 Check
PLAT432_ALERT_2_G Short Inter X...Y Contact  O8       ..C16B     .       2.78 Ang.
                                                      x,y,z  =     1_555 Check
PLAT432_ALERT_2_G Short Inter X...Y Contact  O8       ..C30B     .       2.78 Ang.
                                            1-x,1/2+y,1/2-z  =     2_655 Check
PLAT432_ALERT_2_G Short Inter X...Y Contact  O16B     ..C15A     .       2.95 Ang.
                                                      x,y,z  =     1_555 Check
PLAT432_ALERT_2_G Short Inter X...Y Contact  O16B     ..C7B      .       3.01 Ang.
                                           1-x,-1/2+y,1/2-z  =     2_645 Check
PLAT790_ALERT_4_G Centre of Gravity not Within Unit Cell: Resd.  #          4 Note
             O
PLAT811_ALERT_5_G No ADDSYM Analysis: Too Many Excluded Atoms ....          ! Info
```

```
PLAT910_ALERT_3_G Missing # of FCF Reflection(s) Below Theta(Min).          1 Note
PLAT912_ALERT_4_G Missing # of FCF Reflections Above STh/L=  0.600       1778 Note
PLAT933_ALERT_2_G Number of HKL-OMIT Records in Embedded .res File         16 Note
PLAT941_ALERT_3_G Average HKL Measurement Multiplicity ..........         3.0 Low
PLAT961_ALERT_5_G Dataset Contains no Negative Intensities .......     Please Check
PLAT965_ALERT_2_G The SHELXL WEIGHT Optimisation has not Converged     Please Check
PLAT978_ALERT_2_G Number C-C Bonds with Positive Residual Density.          0 Info
PLAT992_ALERT_5_G Repd & Actual _reflns_number_gt Values Differ by          2 Check
```

```
   3 ALERT level A = Most likely a serious problem - resolve or explain
  19 ALERT level B = A potentially serious problem, consider carefully
  24 ALERT level C = Check. Ensure it is not caused by an omission or oversight
 154 ALERT level G = General information/check it is not something unexpected

   3 ALERT type 1 CIF construction/syntax error, inconsistent or missing data
  62 ALERT type 2 Indicator that the structure model may be wrong or deficient
   8 ALERT type 3 Indicator that the structure quality may be low
 124 ALERT type 4 Improvement, methodology, query or suggestion
   3 ALERT type 5 Informative message, check
```

Datablock: gea361

```
Bond precision:    C-C = 0.0052 A           Wavelength=0.71073

Cell:              a=11.6193(2)     b=9.5705(2)        c=27.0503(5)
                   alpha=90         beta=97.641(2)     gamma=90
Temperature:       130 K

                  Calculated                         Reported
Volume            2981.35(10)                        2981.35(10)
Space group       P 21/n                             P 21/n
Hall group        -P 2yn                             -P 2yn
Moiety formula    C30 H23 Cs2 O10 Ta                 ?
Sum formula       C30 H23 Cs2 O10 Ta                 C30 H23 Cs2 O10 Ta
Mr                990.25                             990.25
Dx,g cm-3         2.206                              2.206
Z                 4                                  4
Mu (mm-1)         6.151                              6.151
F000              1864.0                             1864.0
F000'             1859.16
h,k,lmax          16,13,37                           16,12,37
Nref              8344                               7332
Tmin,Tmax         0.126,0.158                        0.818,1.000
Tmin'             0.040
```

Correction method= # Reported T Limits: Tmin=0.818 Tmax=1.000
AbsCorr = MULTI-SCAN

Data completeness= 0.879 Theta(max)= 29.569

R(reflections)= 0.0273(6463) wR2(reflections)= 0.0540(7332)

S = 1.065 Npar= 403

The following ALERTS were generated. Each ALERT has the format
test-name_ALERT_alert-type_alert-level.
Click on the hyperlinks for more details of the test.

Alert level B

```
PLAT910_ALERT_3_B Missing # of FCF Reflection(s) Below Theta(Min).        14 Note
```

Author Response: SCXRD experiment completness is 99.7%.

Alert level C

```
PLAT048_ALERT_1_C MoietyFormula Not Given (or Incomplete) ........     Please Check
PLAT411_ALERT_2_C Short Inter H...H Contact  H10A     ..H27A     .       2.12 Ang.
                                        1/2-x,-1/2+y,1/2-z  =      2_545 Check
PLAT906_ALERT_3_C Large K Value in the Analysis of Variance ......      2.561 Check
PLAT911_ALERT_3_C Missing FCF Refl Between Thmin & STh/L=    0.600         6 Report
PLAT975_ALERT_2_C Check Calcd Resid. Dens.  0.97Ang From O10     .       1.01 eA-3
PLAT977_ALERT_2_C Check Negative Difference Density on H21A      .      -0.32 eA-3
```

Alert level G

```
PLAT004_ALERT_5_G Polymeric Structure Found with Maximum Dimension         2 Info
PLAT083_ALERT_2_G SHELXL Second Parameter in WGHT  Unusually Large      7.04 Why ?
PLAT164_ALERT_4_G Nr. of Refined C-H H-Atoms in Heavy-Atom Struct.         2 Note
PLAT343_ALERT_2_G Unusual          Angle Range in Main Residue for        C7 Check
PLAT343_ALERT_2_G Unusual          Angle Range in Main Residue for        C8 Check
PLAT343_ALERT_2_G Unusual          Angle Range in Main Residue for       C25 Check
PLAT343_ALERT_2_G Unusual          Angle Range in Main Residue for       C26 Check
PLAT343_ALERT_2_G Unusual          Angle Range in Main Residue for       C27 Check
PLAT764_ALERT_4_G Overcomplete CIF Bond List Detected (Rep/Expd) .      1.22 Ratio
PLAT774_ALERT_1_G Check   X-Y Bond in CIF: Ta1      --Cs2        ..     4.47 Ang.
PLAT779_ALERT_4_G Suspect or Irrelevant (Bond) Angle(s) in CIF ...     44.56 Deg.
              O3   -C7   -CS2     1_555   1_555   1_555 ......     #  252 Check
PLAT779_ALERT_4_G Suspect or Irrelevant (Bond) Angle(s) in CIF ...     41.00 Deg.
              CS2  -C8   -H8      1_555   1_555   1_555 ......     #  265 Check
PLAT779_ALERT_4_G Suspect or Irrelevant (Bond) Angle(s) in CIF ...     34.96 Deg.
              O9   -C25  -CS1     1_555   1_555   1_555 ......     #  350 Check
PLAT794_ALERT_5_G Tentative Bond Valency for Ta1       (V)       .      5.06 Info
PLAT912_ALERT_4_G Missing # of FCF Reflections Above STh/L=  0.600       952 Note
PLAT913_ALERT_3_G Missing # of Very Strong Reflections in FCF ....         2 Note
PLAT933_ALERT_2_G Number of HKL-OMIT Records in Embedded .res File         7 Note
```

```
PLAT941_ALERT_3_G Average HKL Measurement Multiplicity ...........       3.7 Low
PLAT961_ALERT_5_G Dataset Contains no Negative Intensities .......    Please Check
PLAT978_ALERT_2_G Number C-C Bonds with Positive Residual Density.         1 Info
```

---

   0 **ALERT level A** = Most likely a serious problem - resolve or explain
   1 **ALERT level B** = A potentially serious problem, consider carefully
   6 **ALERT level C** = Check. Ensure it is not caused by an omission or oversight
  20 **ALERT level G** = General information/check it is not something unexpected

   2 ALERT type 1 CIF construction/syntax error, inconsistent or missing data
  11 ALERT type 2 Indicator that the structure model may be wrong or deficient
   5 ALERT type 3 Indicator that the structure quality may be low
   6 ALERT type 4 Improvement, methodology, query or suggestion
   3 ALERT type 5 Informative message, check

Datablock: gea279

Bond precision: C-C = 0.0154 A   Wavelength=0.71073

Cell:	a=21.7060(3)	b=13.4010(2)	c=47.338(1)
	alpha=90	beta=102.960(2)	gamma=90
Temperature:	296 K		

	Calculated	Reported
Volume	13419.0(4)	13419.0(4)
Space group	I 2/a	I 2/a
Hall group	-I 2ya	-I 2ya
Moiety formula	2(C108 H72 Cs9 O45.60 Ta6), 5(O0.50), 4(O0.50), 2.8(O)	?
Sum formula	C216 H144 Cs18 O99 Ta12	C36 H24 Cs3 O16.50 Ta2
Mr	8887.10	1481.18
Dx,g cm-3	2.200	2.199
Z	2	12
Mu (mm-1)	7.360	7.360
F000	8196.0	8196.0
F000’	8170.70	
h,k,lmax	29,18,64	29,18,60
Nref	17448	15139
Tmin,Tmax	0.186,0.413	0.593,1.000
Tmin’	0.135	

Correction method= # Reported T Limits: Tmin=0.593 Tmax=1.000
AbsCorr = MULTI-SCAN

Data completeness= 0.868 Theta(max)= 28.760

R(reflections)= 0.0522(10845) wR2(reflections)= 0.1520(15139)

S = 1.075 Npar= 791

The following ALERTS were generated. Each ALERT has the format
test-name_ALERT_alert-type_alert-level.
Click on the hyperlinks for more details of the test.

Alert level A

```
PLAT973_ALERT_2_A Check Calcd Positive Resid. Density on       Ta1       2.09 eA-3
```

Author Response: Residual density without any chemical sense

Alert level B

```
PLAT430_ALERT_2_B Short Inter D...A Contact  O3W      ..O4W      .       2.77 Ang.
                                                 x,y,z  =      1_555 Check
PLAT910_ALERT_3_B Missing # of FCF Reflection(s) Below Theta(Min).         28 Note
```

Author Response: XRD experiment completeness is 99.6%

```
PLAT934_ALERT_3_B Number of (Iobs-Icalc)/Sigma(W) > 10 Outliers ..          7 Check
PLAT971_ALERT_2_B Check Calcd Resid. Dens.  0.95Ang From Cs4             3.17 eA-3
```

Author Response: Residual density without any sense

```
PLAT973_ALERT_2_B Check Calcd Positive Resid. Density on       Ta2       1.95 eA-3
```

Author Response: Residual density without any chemical sense

```
PLAT975_ALERT_2_B Check Calcd Resid. Dens.  0.72Ang From O1W     .       1.53 eA-3
PLAT975_ALERT_2_B Check Calcd Resid. Dens.  0.70Ang From O2W     .       1.53 eA-3
PLAT975_ALERT_2_B Check Calcd Resid. Dens.  0.61Ang From O2W     .       1.52 eA-3
```

Alert level C

```
PLAT048_ALERT_1_C MoietyFormula Not Given (or Incomplete) ........     Please Check
PLAT202_ALERT_3_C Isotropic non-H Atoms in Anion/Solvent .........          1 Check
              O9W
PLAT220_ALERT_2_C NonSolvent   Resd 1   O   Ueq(max)/Ueq(min) Range        3.2 Ratio
PLAT241_ALERT_2_C High   'MainMol' Ueq as Compared to Neighbors of        Cs1 Check
PLAT242_ALERT_2_C Low    'MainMol' Ueq as Compared to Neighbors of         O6 Check
PLAT242_ALERT_2_C Low    'MainMol' Ueq as Compared to Neighbors of        O10 Check
PLAT242_ALERT_2_C Low    'MainMol' Ueq as Compared to Neighbors of        O14 Check
PLAT242_ALERT_2_C Low    'MainMol' Ueq as Compared to Neighbors of        O19 Check
```

```
PLAT342_ALERT_3_C Low Bond Precision on  C-C Bonds ...............    0.01544 Ang.
PLAT906_ALERT_3_C Large K Value in the Analysis of Variance ......      4.924 Check
PLAT911_ALERT_3_C Missing FCF Refl Between Thmin & STh/L=    0.600         15 Report
PLAT971_ALERT_2_C Check Calcd Resid. Dens.  0.77Ang From Cs4             2.25 eA-3
```

Author Response: Residual density without any sense

```
PLAT971_ALERT_2_C Check Calcd Resid. Dens.  0.88Ang From Ta1             1.85 eA-3
```

Author Response: Residual density without any sense

```
PLAT971_ALERT_2_C Check Calcd Resid. Dens.  0.67Ang From Cs7             1.81 eA-3
```

Author Response: Residual density without any sense

```
PLAT971_ALERT_2_C Check Calcd Resid. Dens.  0.82Ang From Ta2             1.79 eA-3
```

Author Response: Residual density without any sense

```
PLAT971_ALERT_2_C Check Calcd Resid. Dens.  0.38Ang From Cs4             1.67 eA-3
```

Author Response: Residual density without any sense

```
PLAT971_ALERT_2_C Check Calcd Resid. Dens.  0.72Ang From O1W             1.53 eA-3
```

Author Response: Residual density without any sense

```
PLAT971_ALERT_2_C Check Calcd Resid. Dens.  0.70Ang From O2W             1.53 eA-3
```

Author Response: Residual density without any sense

```
PLAT971_ALERT_2_C Check Calcd Resid. Dens.  0.61Ang From O2W             1.52 eA-3
```

Author Response: Residual density without any sense

```
PLAT972_ALERT_2_C Check Calcd Resid. Dens.  0.59Ang From Cs1            -2.37 eA-3
PLAT972_ALERT_2_C Check Calcd Resid. Dens.  0.69Ang From Cs1            -2.06 eA-3
PLAT972_ALERT_2_C Check Calcd Resid. Dens.  0.51Ang From Cs1            -2.03 eA-3
PLAT972_ALERT_2_C Check Calcd Resid. Dens.  0.85Ang From Ta3            -1.62 eA-3
PLAT973_ALERT_2_C Check Calcd Positive Resid. Density on       Ta3       1.47 eA-3
```

Author Response: Residual density without any chemical sense

```
PLAT975_ALERT_2_C Check Calcd Resid. Dens.  0.72Ang From O5W     .       1.40 eA-3
PLAT975_ALERT_2_C Check Calcd Resid. Dens.  0.72Ang From O5W     .       1.33 eA-3
PLAT975_ALERT_2_C Check Calcd Resid. Dens.  0.87Ang From O10W    .       1.24 eA-3
PLAT975_ALERT_2_C Check Calcd Resid. Dens.  0.63Ang From O7W     .       1.24 eA-3
PLAT975_ALERT_2_C Check Calcd Resid. Dens.  0.73Ang From O1W     .       1.22 eA-3
PLAT975_ALERT_2_C Check Calcd Resid. Dens.  0.80Ang From O8W     .       1.08 eA-3
PLAT975_ALERT_2_C Check Calcd Resid. Dens.  0.69Ang From O9W     .       1.07 eA-3
PLAT977_ALERT_2_C Check Negative Difference Density on H12       .      -0.44 eA-3
```

Alert level G

```
PLAT004_ALERT_5_G Polymeric Structure Found with Maximum Dimension          3 Info
PLAT045_ALERT_1_G Calculated and Reported Z Differ by a Factor ...      0.167 Check
PLAT180_ALERT_4_G Check Cell Rounding: # of Values Ending with 0 =          3 Note
PLAT300_ALERT_4_G Atom Site Occupancy of Cs3         Constrained at      0.85 Check
PLAT300_ALERT_4_G Atom Site Occupancy of Cs4         Constrained at      0.85 Check
PLAT300_ALERT_4_G Atom Site Occupancy of Cs5         Constrained at       0.3 Check
PLAT300_ALERT_4_G Atom Site Occupancy of Cs6         Constrained at       0.3 Check
PLAT300_ALERT_4_G Atom Site Occupancy of Cs7         Constrained at       0.2 Check
PLAT300_ALERT_4_G Atom Site Occupancy of O1W         Constrained at       0.5 Check
PLAT300_ALERT_4_G Atom Site Occupancy of O2W         Constrained at       0.5 Check
PLAT300_ALERT_4_G Atom Site Occupancy of O8W         Constrained at       0.5 Check
PLAT300_ALERT_4_G Atom Site Occupancy of O10W        Constrained at       0.4 Check
PLAT300_ALERT_4_G Atom Site Occupancy of O11W        Constrained at       0.4 Check
PLAT300_ALERT_4_G Atom Site Occupancy of O4W         Constrained at       0.5 Check
PLAT300_ALERT_4_G Atom Site Occupancy of O5W         Constrained at       0.5 Check
PLAT300_ALERT_4_G Atom Site Occupancy of O7W         Constrained at       0.5 Check
PLAT300_ALERT_4_G Atom Site Occupancy of O9W         Constrained at       0.7 Check
PLAT301_ALERT_3_G Main Residue  Disorder .............(Resd  1  )         6% Note
PLAT302_ALERT_4_G Anion/Solvent/Minor-Residue Disorder (Resd  2  )       100% Note
PLAT302_ALERT_4_G Anion/Solvent/Minor-Residue Disorder (Resd  3  )       100% Note
PLAT302_ALERT_4_G Anion/Solvent/Minor-Residue Disorder (Resd  4  )       100% Note
PLAT302_ALERT_4_G Anion/Solvent/Minor-Residue Disorder (Resd  5  )       100% Note
PLAT311_ALERT_2_G Isolated Disordered Oxygen Atom (No H's ?) .....        O4W Check
PLAT311_ALERT_2_G Isolated Disordered Oxygen Atom (No H's ?) .....        O5W Check
PLAT311_ALERT_2_G Isolated Disordered Oxygen Atom (No H's ?) .....        O7W Check
PLAT311_ALERT_2_G Isolated Disordered Oxygen Atom (No H's ?) .....        O9W Check
PLAT343_ALERT_2_G Unusual          Angle Range in Main Residue for        C14 Check
PLAT343_ALERT_2_G Unusual          Angle Range in Main Residue for        C15 Check
PLAT764_ALERT_4_G Overcomplete CIF Bond List Detected (Rep/Expd) .       1.46 Ratio
PLAT774_ALERT_1_G Check   X-Y Bond in CIF: Ta1      --Cs5       ..       4.07 Ang.
PLAT774_ALERT_1_G Check   X-Y Bond in CIF: Ta1      --Cs7       ..       4.10 Ang.
PLAT774_ALERT_1_G Check   X-Y Bond in CIF: Ta1      --Cs1       ..       4.44 Ang.
PLAT774_ALERT_1_G Check   X-Y Bond in CIF: Ta1      --Cs1       ..       4.45 Ang.
PLAT774_ALERT_1_G Check   X-Y Bond in CIF: Ta2      --Cs1       ..       4.12 Ang.
PLAT774_ALERT_1_G Check   X-Y Bond in CIF: Ta2      --Cs7       ..       4.14 Ang.
PLAT774_ALERT_1_G Check   X-Y Bond in CIF: Ta2      --Cs5       ..       4.16 Ang.
PLAT774_ALERT_1_G Check   X-Y Bond in CIF: Ta2      --Cs2       ..       4.31 Ang.
PLAT774_ALERT_1_G Check   X-Y Bond in CIF: Ta3      --Cs5       ..       4.13 Ang.
PLAT774_ALERT_1_G Check   X-Y Bond in CIF: Ta3      --Cs6       ..       4.20 Ang.
PLAT774_ALERT_1_G Check   X-Y Bond in CIF: Ta3      --Cs3       ..       4.50 Ang.
PLAT779_ALERT_4_G Suspect or Irrelevant (Bond) Angle(s) in CIF ...      34.80 Deg.
              O5   -C7   -CS5     1_555   1_555   1_555 ......     #  741 Check
PLAT779_ALERT_4_G Suspect or Irrelevant (Bond) Angle(s) in CIF ...      41.00 Deg.
              O6   -C13  -CS5     1_555   1_555   1_555 ......     #  785 Check
PLAT779_ALERT_4_G Suspect or Irrelevant (Bond) Angle(s) in CIF ...      41.60 Deg.
              CS6  -C18  -H18     1_555   1_555   1_555 ......     #  831 Check
```

```
PLAT779_ALERT_4_G Suspect or Irrelevant (Bond) Angle(s) in CIF ...      43.00 Deg.
              O1   -C19  -CS7     1_555   1_555   1_555 ......     #  838 Check
PLAT779_ALERT_4_G Suspect or Irrelevant (Bond) Angle(s) in CIF ...      44.90 Deg.
              O11  -C25  -CS7     1_555   1_555   1_555 ......     #  877 Check
PLAT779_ALERT_4_G Suspect or Irrelevant (Bond) Angle(s) in CIF ...      40.90 Deg.
              O13  -C36  -CS5     1_555   1_555   1_555 ......     #  939 Check
PLAT779_ALERT_4_G Suspect or Irrelevant (Bond) Angle(s) in CIF ...      43.70 Deg.
              O14  -C37  -CS5     1_555   1_555   1_555 ......     #  949 Check
PLAT779_ALERT_4_G Suspect or Irrelevant (Bond) Angle(s) in CIF ...      44.20 Deg.
              CS3  -C41  -H41     5_666   1_555   1_555 ......     #  997 Check
PLAT779_ALERT_4_G Suspect or Irrelevant (Bond) Angle(s) in CIF ...      44.00 Deg.
              O15  -C42  -CS3     1_555   1_555   5_666 ......     # 1004 Check
PLAT780_ALERT_1_G Coordinates do not Form a Properly Connected Set     Please Do !
PLAT794_ALERT_5_G Tentative Bond Valency for Ta1       (V)       .       5.05 Info
PLAT794_ALERT_5_G Tentative Bond Valency for Ta2       (V)       .       4.97 Info
PLAT794_ALERT_5_G Tentative Bond Valency for Ta3       (V)       .       5.01 Info
PLAT912_ALERT_4_G Missing # of FCF Reflections Above STh/L=  0.600       2190 Note
PLAT913_ALERT_3_G Missing # of Very Strong Reflections in FCF ....          3 Note
PLAT933_ALERT_2_G Number of HKL-OMIT Records in Embedded .res File         33 Note
PLAT941_ALERT_3_G Average HKL Measurement Multiplicity ..........         1.9 Low
PLAT952_ALERT_5_G Calculated (ThMax) and CIF-Reported Lmax Differ.          4 Units
PLAT958_ALERT_1_G Calculated (ThMax) and Actual (FCF) Lmax Differ.          4 Units
PLAT961_ALERT_5_G Dataset Contains no Negative Intensities .......     Please Check
PLAT978_ALERT_2_G Number C-C Bonds with Positive Residual Density.          0 Info
```

---

1 **ALERT level A** = Most likely a serious problem - resolve or explain
8 **ALERT level B** = A potentially serious problem, consider carefully
32 **ALERT level C** = Check. Ensure it is not caused by an omission or oversight
61 **ALERT level G** = General information/check it is not something unexpected

15 ALERT type 1 CIF construction/syntax error, inconsistent or missing data
42 ALERT type 2 Indicator that the structure model may be wrong or deficient
9 ALERT type 3 Indicator that the structure quality may be low
30 ALERT type 4 Improvement, methodology, query or suggestion
6 ALERT type 5 Informative message, check

---

checkCIF publication errors

Alert level A

```
PUBL004_ALERT_1_A  The contact author's name and address are missing,
            _publ_contact_author_name and _publ_contact_author_address.
PUBL005_ALERT_1_A  _publ_contact_author_email, _publ_contact_author_fax and
            _publ_contact_author_phone are all missing.
            At least one of these should be present.
PUBL006_ALERT_1_A  _publ_requested_journal is missing
            e.g. 'Acta Crystallographica Section C'
PUBL008_ALERT_1_A  _publ_section_title is missing. Title of paper.
PUBL009_ALERT_1_A  _publ_author_name is missing. List of author(s) name(s).
PUBL010_ALERT_1_A  _publ_author_address is missing. Author(s) address(es).
PUBL012_ALERT_1_A  _publ_section_abstract is missing.
            Abstract of paper in English.
```

Alert level G

```
PUBL017_ALERT_1_G The _publ_section_references section is missing or
            empty.
```

```
   7 ALERT level A = Data missing that is essential or data in wrong format
   1 ALERT level G = General alerts. Data that may be required is missing
```

Publication of your CIF

You should attempt to resolve as many as possible of the alerts in all categories. Often the minor alerts point to easily fixed oversights, errors and omissions in your CIF or refinement strategy, so attention to these fine details can be worthwhile. In order to resolve some of the more serious problems it may be necessary to carry out additional measurements or structure refinements. However, the nature of your study may justify the reported deviations from journal submission requirements and the more serious of these should be commented upon in the discussion or experimental section of a paper or in the "special_details" fields of the CIF. *checkCIF* was carefully designed to identify outliers and unusual parameters, but every test has its limitations and alerts that are not important in a particular case may appear. Conversely, the absence of alerts does not guarantee there are no aspects of the results needing attention. It is up to the individual to critically assess their own results and, if necessary, seek expert advice.

If level A alerts remain, which you believe to be justified deviations, and you intend to submit this CIF for publication in a journal, you should additionally insert an explanation in your CIF using the Validation Reply Form (VRF) below. This will allow your explanation to be considered as part of the review process.

Validation response form

Please find below a validation response form (VRF) that can be filled in and pasted into your CIF.

```
# start Validation Reply Form
_vrf_PUBL004_GLOBAL
;
PROBLEM: The contact author’s name and address are missing,
RESPONSE: ...
;
_vrf_PUBL005_GLOBAL
;
PROBLEM: _publ_contact_author_email, _publ_contact_author_fax and
RESPONSE: ...
;
_vrf_PUBL006_GLOBAL
;
PROBLEM: _publ_requested_journal is missing
RESPONSE: ...
;
_vrf_PUBL008_GLOBAL
;
PROBLEM: _publ_section_title is missing. Title of paper.
RESPONSE: ...
```

```
;
_vrf_PUBL009_GLOBAL
;
PROBLEM: _publ_author_name is missing. List of author(s) name(s).
RESPONSE: ...
;
_vrf_PUBL010_GLOBAL
;
PROBLEM: _publ_author_address is missing. Author(s) address(es).
RESPONSE: ...
;
_vrf_PUBL012_GLOBAL
;
PROBLEM: _publ_section_abstract is missing.
RESPONSE: ...
;
# end Validation Reply Form
```

If you wish to submit your CIF for publication in Acta Crystallographica Section C or E, you should upload your CIF via the web. If you wish to submit your CIF for publication in IUCrData you should upload your CIF via the web. If your CIF is to form part of a submission to another IUCr journal, you will be asked, either during electronic submission or by the Co-editor handling your paper, to upload your CIF via our web site.

PLATON version of 10/05/2023; check.def file version of 10/05/2023

Datablock gea203 - ellipsoid plot

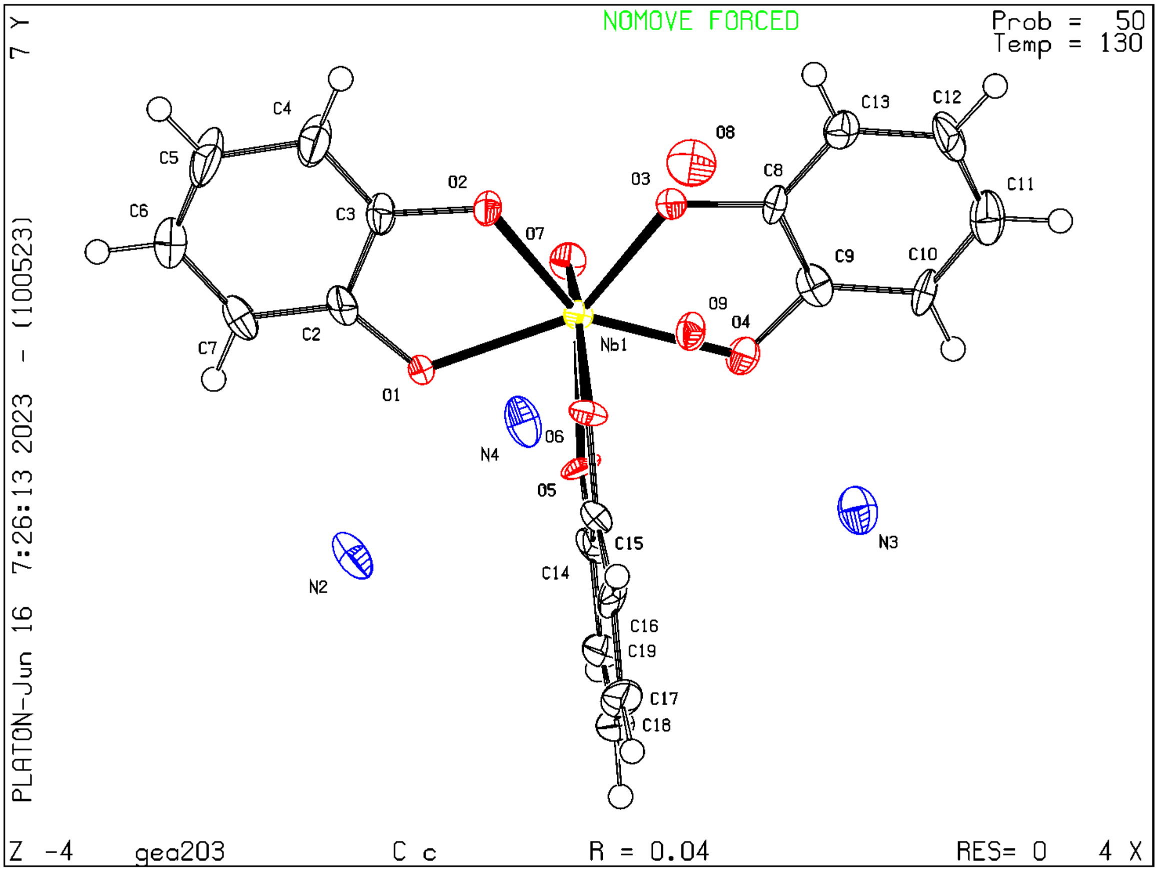

Datablock gea381 - ellipsoid plot

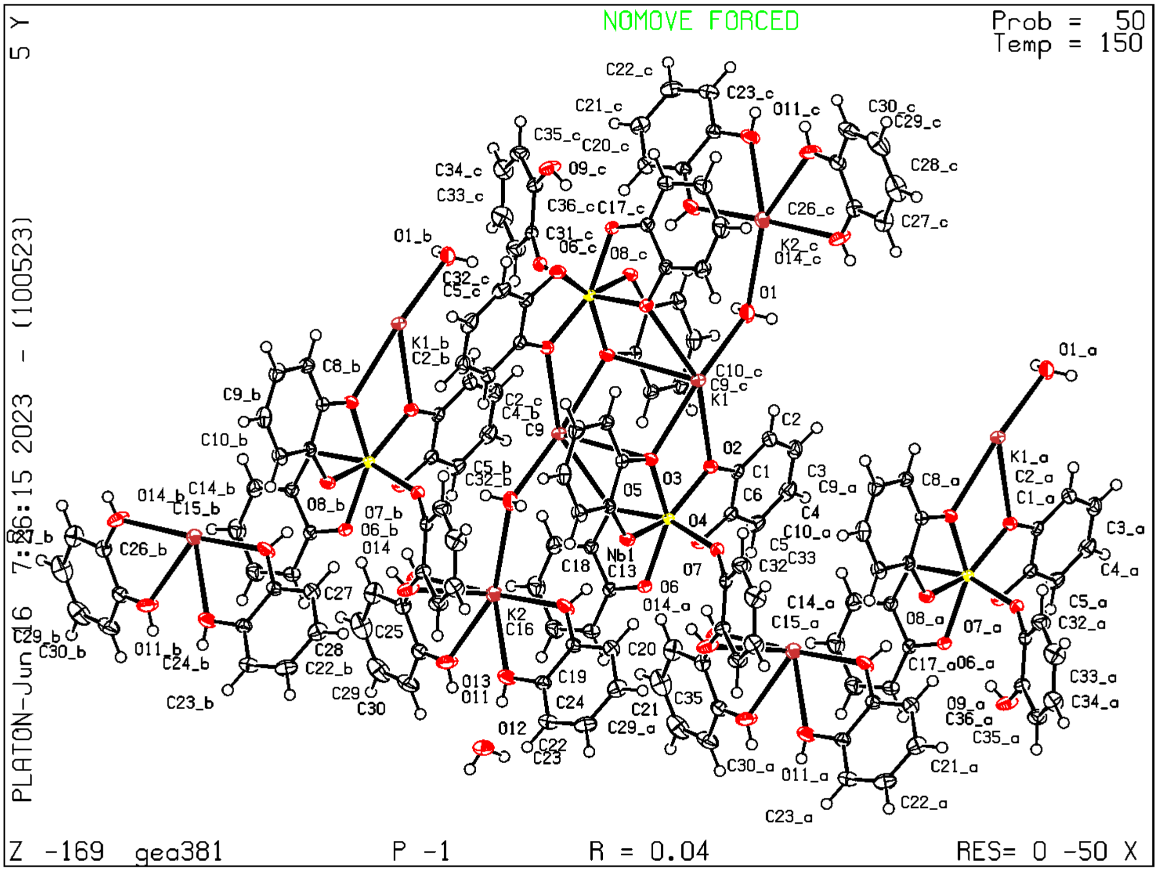

Datablock gea354 - ellipsoid plot

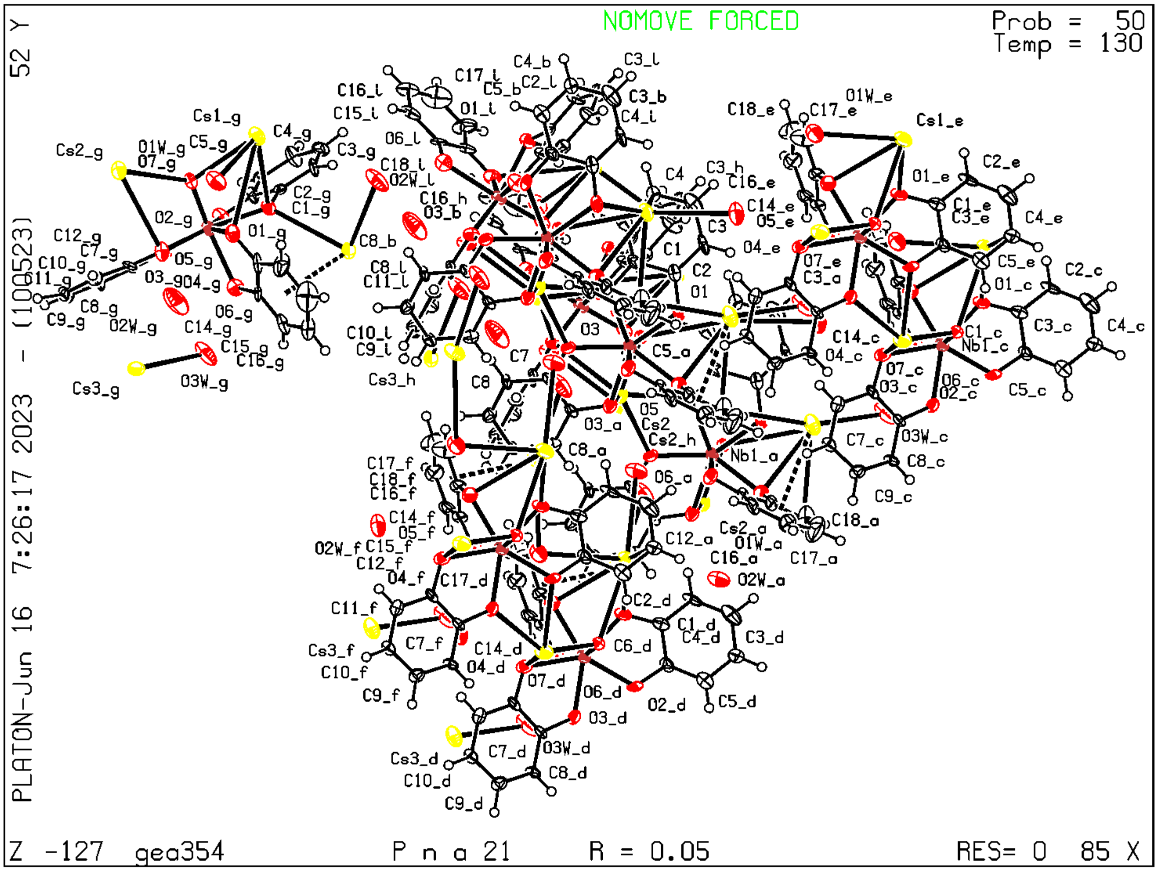

Datablock gea355 - ellipsoid plot

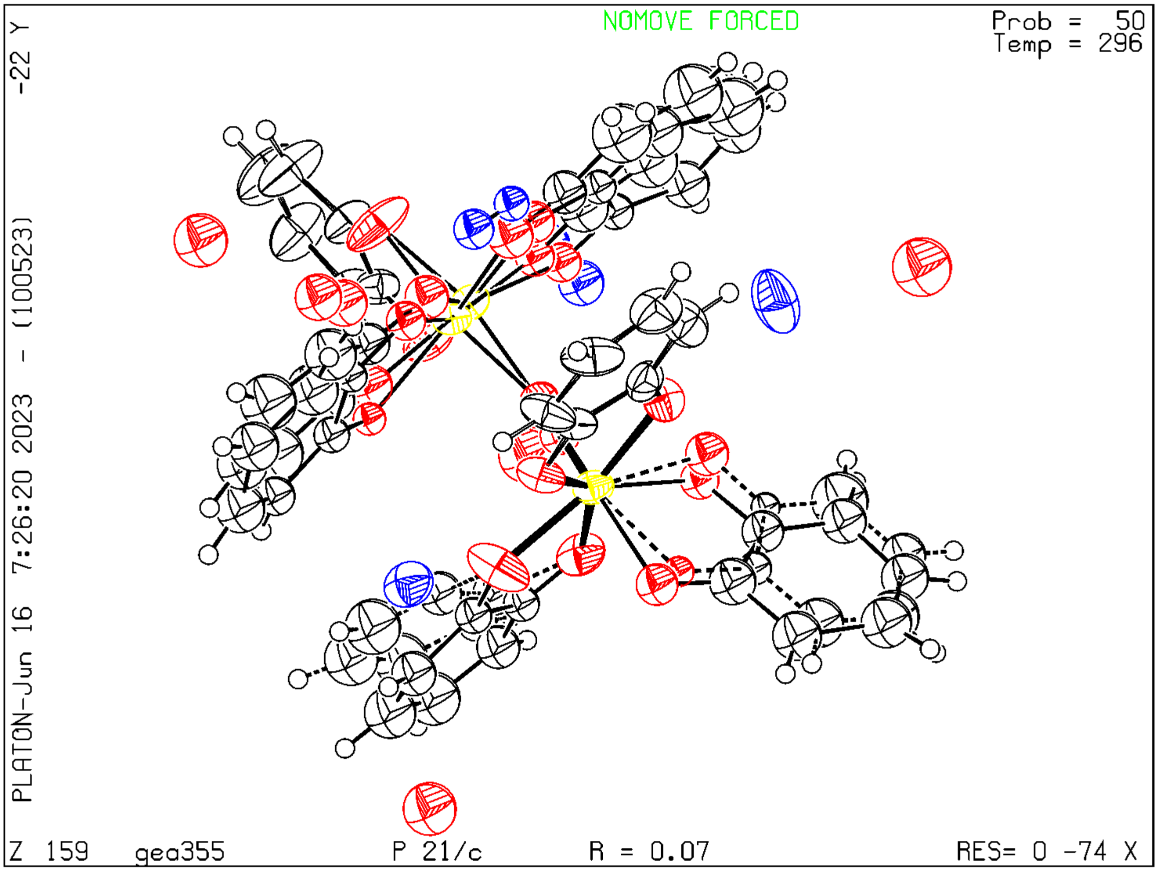

Datablock gea361 - ellipsoid plot

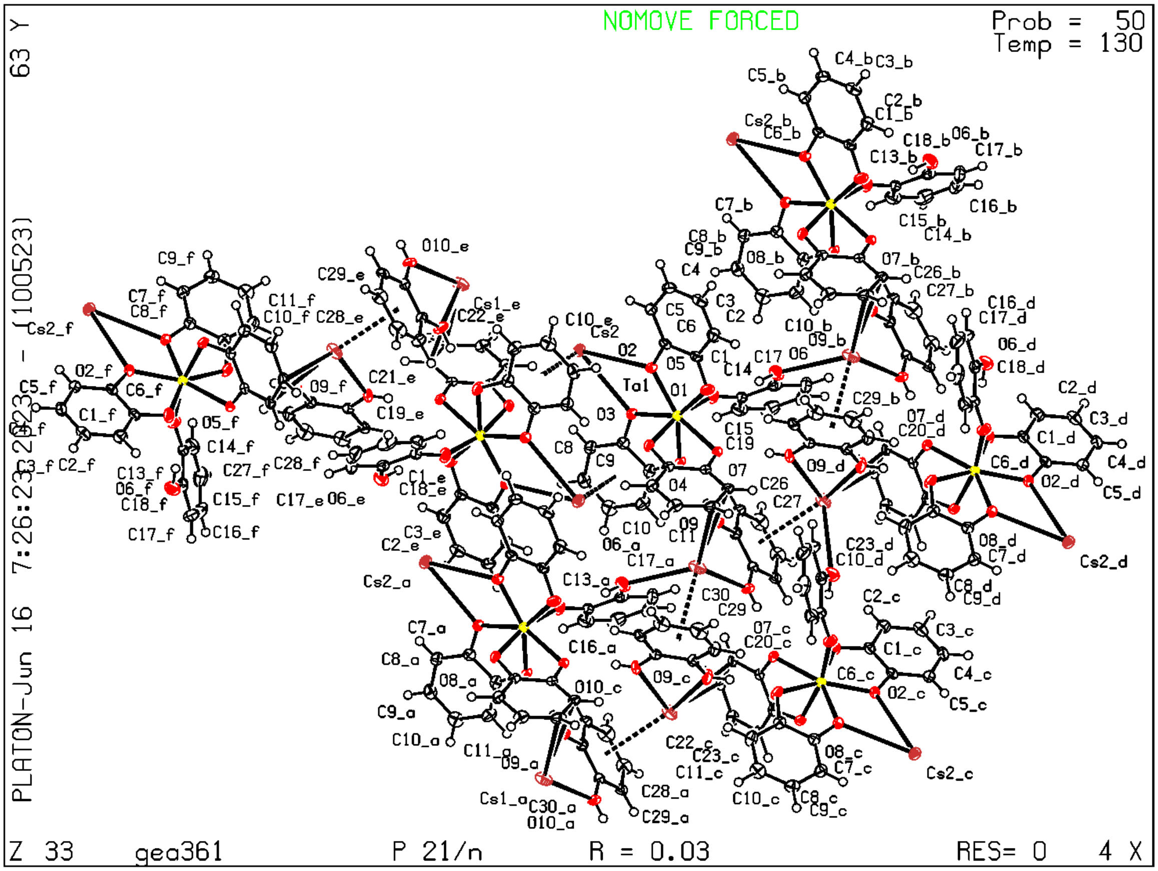

Datablock gea279 - ellipsoid plot

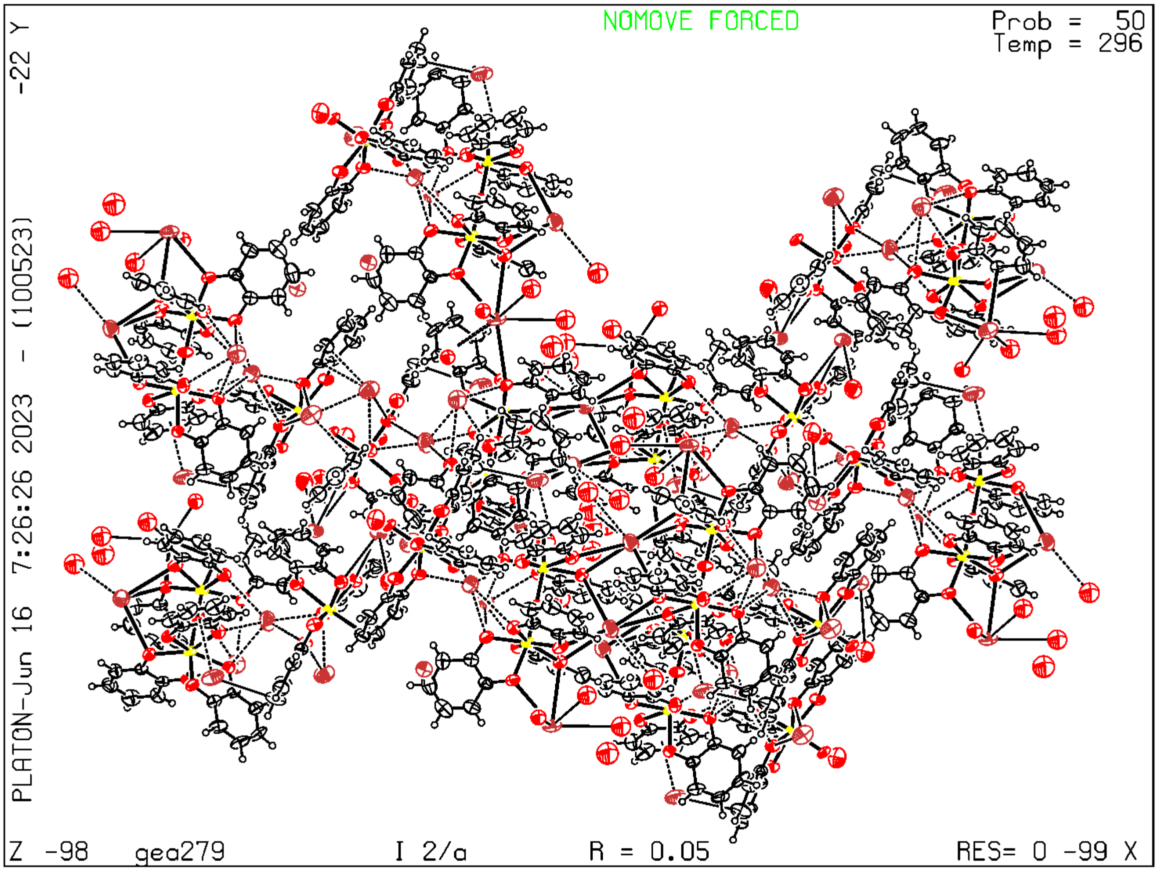

Supplement: Supplementary file 1 [file molecules-28-04912-s001.zip › checkcif.pdf]
