# Supplementary material for: Not So Similar: Different Ways of Nb(V) and Ta(V) Catecholate Complexation
Source: Molecules. 2023 Jun 22;28(13):4912. doi: 10.3390/molecules28134912 (PMC10361208; doi:10.3390/molecules28134912)
Supplement: Supplementary file 1 [file molecules-28-04912-s001.zip › molecules-2457410-supplementary tables and figures.pdf]

Not So Similar: Different Ways of Nb(V) and Ta(V) Catecholate Complexation

Pavel A. Abramov [1,2,*] and Maxim N. Sokolov [1]

[1] Nikolaev Institute of Inorganic Chemistry SB RAS, 3 Akad. Lavrentiev Ave., 630090 Novosibirsk, Russia
[2] Research School of Chemistry and Applied Biomedical Sciences, Tomsk Polytechnic University, 634034 Tomsk, Russia
* Correspondence: abramov@niic.nsc.ru

Supporting Information

Table of contents

Table S1. SCXRD Experimental details....3

Table S2. Selected geometric parameters (Å) 5

Figure S1. Powder patterns comparison for **1**: experimental at 298K (black curve), calculated at 130K (red curve). 8

Figure S2. Powder patterns comparison for **4**: experimental at 298K (black curve), calculated at 298K (red curve). 9

Figure S3. Powder patterns comparison for **5**: experimental at 298K (black curve), calculated at 130K (red curve). 9

Figure S4. Powder patterns comparison for **6**: experimental at 298K (black curve), calculated at 296K (red curve). 10

Figure S5. TGA data for **1**....11

Figure S6. TGA data for **2**....11

Figure S7. TGA data for **3**....12

Figure S8. TGA data for **4**....12

Figure S9. TGA data for **5**....13

Figure S10. TGA data for **6**....13

Figure S11. Crystal packing of **1**....14

Figure S12. Crystal packing of **2**....15

Figure S13. Crystal packing of **3**....16

Figure S14. Crystal packing of **5**....17

Figure S15. Crystal packing of **6**....18

Figure S16. Ellipsoid representation of $[NbO(cat)_3]^{3-}$ in the crystal structure of **1**....19

Figure S17. Ellipsoid representation of $[Nb(cat)_3(Hcat)]^{2-}$ in the crystal structure of **2**. 20

Figure S18. Ellipsoid representation of $[NbO(cat)_3]^{3-}$ in the crystal structure of **3**....21

Figure S19. Ellipsoid representation of $[Ta_2O(cat)_6]^{4-}$ in the crystal structure of **4**. 22
Figure S20. Ellipsoid representation of $[Ta(cat)_3(Hcat)]^{2-}$ in the crystal structure of **5**. 23
Figure S21. Ellipsoid representation of $[Ta_2O(cat)_6]^{4-}$ in the crystal structure of **6**. 24

Table S1. SCXRD Experimental details

	**1**	**2**	**3**	**4**
Chemical formula	$C_{18}H_{28}N_3NbO_9$	$C_{36}H_{33}K_2NbO_{14}$	$C_{18}H_{18}Cs_3NbO_{10}$	$C_{36}H_{24}N_4O_{15.35}Ta_2$
M_r	523.34	860.73	885.96	1120.09
Crystal system, space group	Monoclinic, *Cc*	Triclinic, $P\bar{1}$	Orthorhombic, $Pna2_1$	Monoclinic, $P2_1/c$
Temperature (K)	130	150	130	296
a, *b*, *c* (Å)	15.9081 (7), 19.7180 (8), 7.0292 (3)	8.7876 (3), 14.0120 (6), 15.4138 (6)	7.4397 (5), 21.460 (3), 16.1460 (12)	11.4815 (5), 11.8561 (4), 32.1364 (9)
α, β, γ (°)	90, 95.172 (4), 90	82.323 (1), 76.607(1), 76.500 (1)	90, 90, 90	90, 99.974 (1), 90
V (Å^3)	2195.91 (16)	1788.95 (12)	2577.9 (5)	4308.5 (3)
Z	4	2	4	4
μ (mm^{-1})	0.60	0.64	4.69	5.14
Crystal size (mm)	0.30 × 0.05 × 0.05	0.20 × 0.12 × 0.12	0.25 × 0.20 × 0.15	0.25 × 0.09 × 0.07
Diffractometer	New Xcalibur, AtlasS2	Bruker Apex Duo	New Xcalibur, AtlasS2	Bruker Apex Duo
Absorption correction	Multi-scan *CrysAlis PRO* 1.171.38.41 (Rigaku Oxford Diffraction, 2015) Empirical absorption correction using spherical harmonics, implemented in SCALE3 ABSPACK scaling algorithm.	Multi-scan *SADABS* (Bruker-AXS, 2004)	Multi-scan *CrysAlis PRO* 1.171.38.41 (Rigaku Oxford Diffraction, 2015) Empirical absorption correction using spherical harmonics, implemented in SCALE3 ABSPACK scaling algorithm.	Multi-scan *SADABS* (Bruker-AXS, 2004)
T_{min}, T_{max}	0.961, 1.000	0.673, 0.748	0.979, 1.000	0.642, 0.746
No. of measured, independent and observed [$I > 2\sigma(I)$] reflections	5417, 3181, 2912	31063, 16957, 12331	8430, 4446, 3579	35157, 11857, 7085
R_{int}	0.040	0.026	0.044	0.055
θ values (°)	θ_{max} = 25.4, θ_{min} = 3.3	θ_{max} = 38.1, θ_{min} = 1.9	θ_{max} = 29.5, θ_{min} = 3.3	θ_{max} = 31.0, θ_{min} = 1.8
$(\sin\theta/\lambda)_{max}$ (Å^{-1})	0.602	0.869	0.693	0.724
Range of *h*, *k*, *l*	$-19 \le h \le 19$<br>$-18 \le k \le 23$<br>$-8 \le l \le 7$	$-14 \le h \le 12$<br>$-22 \le k \le 23$<br>$-26 \le l \le 24$	$-10 \le h \le 9$<br>$-26 \le k \le 14$<br>$-14 \le l \le 21$	$-16 \le h \le 15$<br>$-16 \le k \le 16$<br>$-44 \le l \le 46$
$R[F^2 > 2\sigma(F^2)]$, $wR(F^2)$, S	0.044, 0.106, 1.03	0.041, 0.087, 1.02	0.051, 0.109, 1.06	0.065, 0.178, 1.03
No. of reflections, parameters, restraints	3181, 280, 2	16959, 505, 0	4446, 289, 13	11857, 485, 0
H-atom treatment	H-atom parameters constrained	H atoms treated by a mixture of independent and constrained refinement	H-atom parameters constrained	H-atom parameters constrained
Weighting scheme	$w = 1/[\sigma^2(F_o^2) + (0.0592P)^2]$ where $P = (F_o^2 + 2F_c^2)/3$	$w = 1/[\sigma^2(F_o^2) + (0.0376P)^2]$ where $P = (F_o^2 + 2F_c^2)/3$	$w = 1/[\sigma^2(F_o^2) + (0.0487P)^2]$ where $P = (F_o^2 + 2F_c^2)/3$	$w = 1/[\sigma^2(F_o^2) + (0.0869P)^2 + 2.2175P]$ where $P = (F_o^2 + 2F_c^2)/3$
$\Delta\rho_{max}$, $\Delta\rho_{min}$ (e Å^{-3})	0.67, -0.74	0.84, -0.79	1.59, -1.48	1.77, -1.79
Absolute structure	Flack x determined using 996 quotients	–	Flack x determined using 976 quotients	–

	[(I+)-(I-)]/[(I+)+(I-)] (Parsons, Flack and Wagner, Acta Cryst. B69 (2013) 249-259).		[(I+)-(I-)]/[(I+)+(I-)] (Parsons, Flack and Wagner, Acta Cryst. B69 (2013) 249-259).	
Absolute structure parameter	-0.15 (4)	–	0.00 (3)	–

	**5**	**6**
Chemical formula	$C_{30}H_{23}Cs_2O_{10}Ta$	$C_{36}H_{24}Cs_3O_{16.50}Ta_2$
M_r	990.25	1481.18
Crystal system, space group	Monoclinic, $P2_1/n$	Monoclinic, $I2/a$
Temperature (K)	130	296
a, b, c (Å)	11.6193 (2), 9.5705 (2), 27.0503 (5)	21.7060 (3), 13.4010 (2), 47.338 (1)
α, β, γ (°)	90, 97.641 (2), 90	90, 102.960 (2), 90
V (Å^3)	2981.35 (10)	13419.0 (4)
Z	4	12
μ (mm^{-1})	6.15	7.52
Crystal size (mm)	0.50 × 0.30 × 0.30	0.26 × 0.20 × 0.12
Diffractometer	New Xcalibur, AtlasS2	New Xcalibur, AtlasS2
Absorption correction	Multi-scan *CrysAlis PRO* 1.171.38.41 (Rigaku Oxford Diffraction, 2015) Empirical absorption correction using spherical harmonics, implemented in SCALE3 ABSPACK scaling algorithm.	Multi-scan *CrysAlis PRO* 1.171.38.41 (Rigaku Oxford Diffraction, 2015) Empirical absorption correction using spherical harmonics, implemented in SCALE3 ABSPACK scaling algorithm.
T_{min}, T_{max}	0.818, 1.000	0.593, 1.000
No. of measured, independent and observed [$I > 2\sigma(I)$] reflections	27096, 7332, 6463	28168, 15139, 10845
R_{int}	0.025	0.038
θ values (°)	$\theta_{max} = 29.6$, $\theta_{min} = 3.4$	$\theta_{max} = 28.8$, $\theta_{min} = 3.3$
$(\sin \theta/\lambda)_{max}$ (Å^{-1})	0.694	0.677
Range of h, k, l	$-15 \le h \le 16$, $-12 \le k \le 12$, $-37 \le l \le 34$	$-13 \le h \le 29$, $-15 \le k \le 18$, $-60 \le l \le 59$
$R[F^2 > 2\sigma(F^2)]$, $wR(F^2)$, S	0.027, 0.054, 1.07	0.056, 0.169, 1.06
No. of reflections, parameters, restraints	7332, 403, 0	15139, 791, 0
H-atom treatment	H atoms treated by a mixture of independent and constrained refinement	H-atom parameters constrained
Weighting scheme	$w = 1/[\sigma^2(F_o^2) + (0.0155P)^2 + 6.9205P]$ where $P = (F_o^2 + 2F_c^2)/3$	$w = 1/[\sigma^2(F_o^2) + (0.0793P)^2]$ where $P = (F_o^2 + 2F_c^2)/3$
$\Delta\rho_{max}$, $\Delta\rho_{min}$ (e Å^{-3})	1.35, -1.04	3.10, -2.39

Computer programs: *CrysAlis PRO* 1.171.38.41 (Rigaku OD, 2015), *APEX2* (Bruker-AXS, 2004), *SAINT* (Bruker-AXS, 2004), *SHELXS2014*/5 (Sheldrick, 2014), *SHELXL2019*/3 (Sheldrick, 2019), ShelXle (Hübschle, 2011), CIFTAB-2014/2 (Sheldrick, 2014).

Table S2. Selected geometric parameters (Å)

**1**			
O1—Nb1	2.131 (6)	O5—Nb1	2.095 (6)
O2—Nb1	2.066 (5)	O6—Nb1	2.148 (5)
O3—Nb1	2.086 (5)	O7—Nb1	1.764 (5)
O4—Nb1	2.200 (7)		
**2**			
O2—Nb1	2.0378 (10)	C7—K1	3.5387 (14)
O3—Nb1	2.0459 (10)	C8—K1^{ii}	3.4759 (15)
O4—Nb1	2.1210 (10)	C13—K2	3.4704 (14)
O5—Nb1	2.0033 (9)	C14—K2	3.3326 (17)
O6—Nb1	2.0873 (10)	C15—K2	3.2072 (18)
O7—Nb1	1.9623 (9)	C16—K2	3.2348 (17)
O8—Nb1	2.1211 (10)	C17—K2	3.4010 (16)
C2—K1^{i}	3.1556 (15)	C18—K2	3.5174 (14)
C3—K1^{i}	3.1435 (15)	C36—K2^{iii}	3.3810 (15)
C7—K1^{ii}	3.1750 (13)		
**3**			
O1—Nb1	2.163 (10)	C2—Cs1	3.816 (15)
O2—Nb1	2.077 (10)	C2—Cs1^{vii}	3.864 (14)
O3—Nb1	2.083 (10)	C2—Cs3^{v}	3.575 (18)
O4—Nb1	2.188 (10)	C3—Cs1^{vii}	3.604 (16)
O5—Nb1	2.121 (10)	C4—Cs1^{vii}	3.378 (19)
O6—Nb1	2.216 (11)	C5—Cs1^{vii}	3.537 (15)
O7—Nb1	1.745 (10)	C5—Cs1^{viii}	3.832 (16)
O1W—Cs1	3.031 (11)	C6—Cs1^{vii}	3.847 (15)
O1W—Cs3^{iv}	3.182 (11)	C6—Cs2^{vii}	3.716 (16)
O1—Cs1	3.047 (9)	C7—Cs2^{viii}	3.459 (14)
O1—Cs3^{v}	3.135 (10)	C7—Cs3	3.672 (13)
O2W—Cs1^{vi}	3.131 (13)	C8—Cs2^{viii}	3.651 (15)
O2W—Cs3	3.745 (16)	C8—Cs3	3.774 (15)
O2—Cs2^{vii}	2.920 (9)	C9—Cs3	3.702 (14)
O2—Cs2^{viii}	3.652 (11)	C10—Cs3	3.537 (14)
O3W—Cs2^{vii}	3.767 (16)	C11—Cs2	3.788 (15)
O3W—Cs3	3.005 (12)	C11—Cs3	3.453 (15)
O3—Cs2^{vii}	3.078 (9)	C12—Cs2	3.482 (13)
O3—Cs2^{viii}	3.131 (10)	C12—Cs3	3.505 (13)
O4—Cs2	3.179 (10)	C13—Cs3^{v}	3.543 (15)
O5—Cs1	3.420 (11)	C14—Cs3^{v}	3.467 (13)
O7—Cs1	3.033 (10)	C15—Cs3^{v}	3.454 (15)
O7—Cs2	2.924 (10)	C16—Cs3^{v}	3.542 (19)
O7—Cs2^{viii}	3.755 (10)	C17—Cs3^{v}	3.57 (2)
C1—Cs1	3.522 (14)	C18—Cs3^{v}	3.600 (19)
C1—Cs3^{v}	3.537 (15)		
**4**			
Ta2B—O7A	2.084 (16)	O4A—Ta1	2.068(17)
Ta2B—O8B	2.057 (16)	O5—Ta1	2.170 (6)
Ta2B—O12B	2.096 (16)	O6—Ta1	2.025 (7)
Ta2B—O13A	1.984 (19)	O9—Ta1	1.881 (5)
Ta2A—O7B	2.095 (13)	O9—Ta2B	1.950 (7)
Ta2A—O8A	2.000 (17)	O9—Ta2A	1.928 (7)
Ta2A—O12A	2.123 (15)	O10—Ta2B	2.100 (7)
Ta2A—O13B	2.006 (18)	O10—Ta2A	2.082 (7)
O1—Ta1	2.088 (7)	O11—Ta2B	2.007 (8)
O2—Ta1	2.053 (5)	O11—Ta2A	2.032 (7)

O3A—Ta1	2.052(13)	O4B—Ta1	2.031(14)
O3B—Ta1	2.20(2)		
**5**			
O1—Ta1	2.086 (2)	C7—Cs2	3.836 (3)
O2—Ta1	2.035 (2)	C8—Cs2^{xi}	3.408 (4)
O3—Ta1	2.030 (2)	C8—Cs2	3.869 (4)
O4—Ta1	2.117 (2)	C9—Cs2^{xi}	3.491 (3)
O5—Ta1	1.936 (2)	C10—Cs2^{xi}	3.577 (4)
O7—Ta1	2.125 (2)	C11—Cs2^{xi}	3.607 (4)
O8—Ta1	1.993 (2)	C12—Cs2^{xi}	3.509 (3)
O2—Cs2	3.097 (2)	C19—Cs1	3.616 (3)
O3—Cs2	3.021 (2)	C20—Cs1	3.350 (4)
O6—Cs1^{ix}	3.140 (3)	C21—Cs1	3.323 (4)
O6—Cs2^{x}	3.458 (3)	C22—Cs1	3.566 (4)
O8—Cs2^{xi}	3.493 (2)	C23—Cs1	3.835 (4)
O9—Cs1	2.903 (3)	C24—Cs1	3.885 (3)
O10—Cs1	3.061 (3)	C25—Cs1^{xii}	3.419 (4)
C1—Cs2^{x}	3.477 (4)	C25—Cs1	3.913 (4)
C2—Cs2^{x}	3.538 (4)	C26—Cs1^{xii}	3.353 (4)
C3—Cs2^{x}	3.551 (4)	C27—Cs1^{xii}	3.385 (4)
C4—Cs2^{x}	3.524 (3)	C28—Cs1^{xii}	3.482 (4)
C5—Cs2^{x}	3.497 (4)	C29—Cs1^{xii}	3.553 (4)
C6—Cs2^{x}	3.468 (3)	C30—Cs1^{xii}	3.553 (4)
C7—Cs2^{xi}	3.375 (3)		
**6**			
O1—Ta1	2.121 (7)	Cs6—Cs6^{xiii}	3.205 (9)
O2—Ta1	2.053 (7)	C1—Cs1	3.841 (11)
O3—Ta1	2.060 (7)	C1—Cs4^{xiv}	3.914 (9)
O4—Ta1	2.030 (7)	C5—Cs4^{xiv}	3.526 (12)
O5—Ta1	2.086 (7)	C6—Cs4^{xiv}	3.497 (12)
O6—Ta1	2.077 (7)	C7—Cs4	3.445 (10)
O7—Ta1	1.899 (6)	C7—Cs5	3.812 (12)
O7—Ta2	1.927 (6)	C8—Cs4	3.557 (10)
O8—Ta2	2.062 (7)	C9—Cs4	3.596 (13)
O9—Ta2	2.111 (7)	C10—Cs4	3.534 (13)
O10—Ta2	2.043 (7)	C11—Cs4	3.476 (13)
O11—Ta2	2.113 (7)	C12—Cs4	3.399 (13)
O12—Ta2	2.060 (7)	C13—Cs2^{xv}	3.501 (10)
O13—Ta2	2.060 (8)	C13—Cs5	3.889 (11)
O14—Ta3	2.045 (7)	C13—Cs7	3.741 (11)
O15—Ta3	2.092 (8)	C14—Cs2^{xv}	3.403 (10)
O16—Ta3	2.037 (7)	C14—Cs4	3.765 (10)
O17—Ta3	2.087 (7)	C15—Cs2^{xv}	3.439 (12)
O18—Ta3^{xiii}	1.9091 (4)	C15—Cs4	3.811 (12)
O18—Ta3	1.9092 (4)	C16—Cs2^{xv}	3.518 (14)
O19—Ta3	2.055 (7)	C17—Cs2^{xv}	3.593 (13)
O20—Ta3	2.112 (7)	C18—Cs2^{xv}	3.600 (12)
O1W—Cs2	3.42 (2)	C18—Cs6	3.774 (13)
O1W—Cs4^{xiv}	3.79 (2)	C19—Cs2^{xv}	3.603 (11)
O1—Cs2^{xv}	3.275 (7)	C19—Cs7	3.867 (13)
O1—Cs7	3.036 (9)	C20—Cs1	3.592 (10)
O2W—Cs2	3.243 (17)	C20—Cs1^{xvi}	3.769 (10)
O2W—Cs4^{xiv}	3.626 (17)	C21—Cs1	3.626 (12)
O2—Cs1^{xvi}	3.046 (7)	C21—Cs1^{xvi}	3.861 (12)
O2—Cs1	3.186 (7)	C24—Cs2^{xv}	3.620 (13)
O3W—Cs3^{xi}	3.415 (12)	C25—Cs7	3.609 (13)

O3—Cs4	3.554 (8)	C26—Cs1	3.562 (11)
O4—Cs1^{xvi}	3.178 (8)	C26—Cs2	3.382 (10)
O5W—Cs7	2.842 (17)	C27—Cs1	3.744 (14)
O5—Cs5	2.815 (8)	C27—Cs2	3.664 (14)
O6—Cs5	3.001 (8)	C30—Cs7	3.799 (16)
O6—Cs7	2.965 (9)	C33—Cs3	3.701 (13)
O7W—Cs5	3.532 (17)	C34—Cs3	3.446 (14)
O7W—Cs6	2.859 (17)	C35—Cs3	3.645 (12)
O7W—Cs7	3.096 (18)	C36—Cs5	3.841 (11)
O7—Cs5	3.700 (8)	C37—Cs5	3.678 (12)
O7—Cs7	3.573 (9)	C37—Cs6^{xiii}	3.287 (12)
O8W—Cs2	3.35 (2)	C37—Cs6	3.740 (11)
O8—Cs1	3.104 (8)	C38—Cs3	3.772 (13)
O9W—Cs3	2.983 (11)	C38—Cs6^{xiii}	3.117 (14)
O9W—Cs5	2.997 (12)	C38—Cs6	3.842 (13)
O9—Cs5	3.159 (8)	C39—Cs3	3.514 (14)
O10W—Cs1	3.25 (3)	C39—Cs6^{xiii}	3.527 (15)
O10W—Cs4^{xvi}	3.22 (3)	C40—Cs3	3.524 (14)
O10—Cs1	3.005 (7)	C41—Cs3	3.718 (14)
O10—Cs2	3.168 (7)	C41—Cs3^{xi}	3.912 (14)
O11W—Cs1^{xvii}	3.37 (3)	C42—Cs3^{xi}	3.818 (11)
O11—Cs7	2.821 (10)	C42—Cs6^{xiii}	3.765 (12)
O12—Cs2	3.184 (7)	C43—Cs3^{xi}	3.554 (10)
O13—Cs5	2.953 (8)	C44—Cs3^{xi}	3.785 (12)
O13—Cs7	3.287 (9)	C45—Cs7^{xviii}	3.534 (13)
O14—Cs5	2.847 (8)	C46—Cs7^{xviii}	3.703 (14)
O14—Cs6	3.037 (8)	C50—Cs3^{xix}	3.486 (12)
O14—Cs6^{xiii}	3.714 (9)	C51—Cs3^{xix}	3.750 (14)
O15—Cs3^{xi}	2.984 (7)	C51—Cs4	3.490 (14)
O16—Cs5	3.712 (8)	C52—Cs4	3.304 (18)
O17—Cs3^{xi}	3.165 (7)	C53—Cs4	3.761 (15)
O19—Cs5	3.167 (8)	C53—Cs6	3.813 (15)
O19—Cs6	2.952 (8)	C54—Cs6	3.703 (11)
Cs5—Cs6	3.798 (5)		

Symmetry code(s): (i) -*x*, -*y*+1, -*z*+2; (ii) -*x*+1, -*y*+1, -*z*+2; (iii) *x*-1, *y*, *z*; (iv) -*x*, -*y*+1, *z*-1/2; (v) -*x*+1, -*y*+1, *z*-1/2; (vi) -*x*, -*y*+1, *z*+1/2; (vii) *x*+1, *y*, *z*; (viii) *x*+1/2, -*y*+3/2, *z*; (ix) *x*, *y*+1, *z*; (x) -*x*+1, -*y*+2, -*z*+1; (xi) -*x*+1, -*y*+1, -*z*+1; (xii) -*x*+3/2, *y*+1/2, -*z*+1/2; (xiii) -*x*+3/2, *y*, -*z*+1; (xiv) *x*-1/2, -*y*+1, *z*; (xv) *x*+1/2, -*y*+2, *z*; (xvi) -*x*+3/2, -*y*+3/2, -*z*+3/2; (xvii) -*x*+1, *y*+1/2, -*z*+3/2; (xviii) *x*, *y*-1, *z*; (xix) *x*+1/2, -*y*+1, *z*.

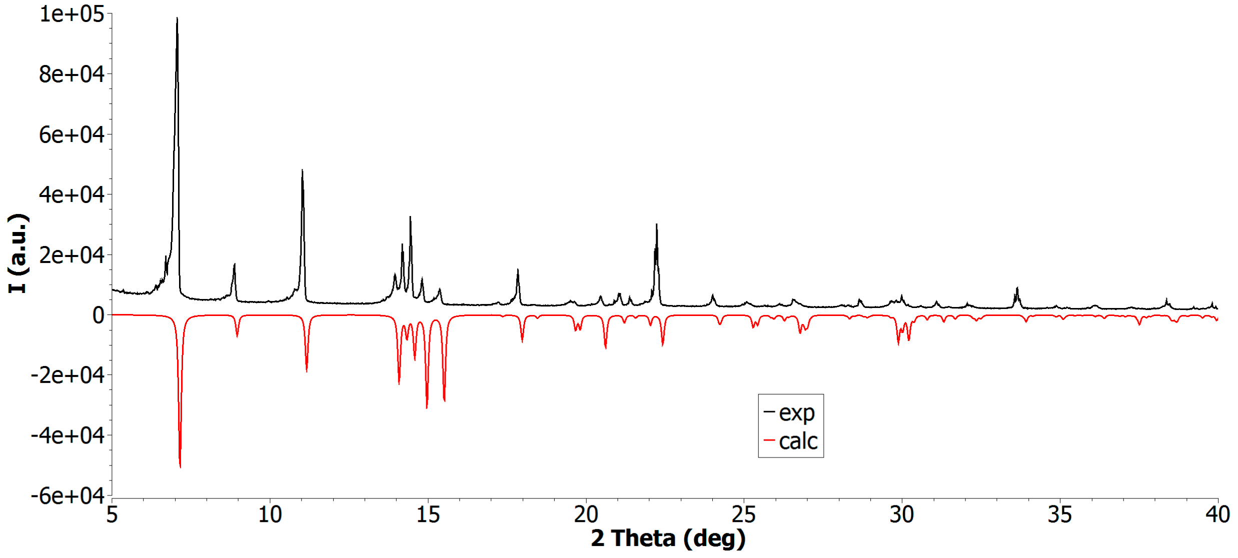

Figure S1. Powder patterns comparison for **1**: experimental at 298K (black curve), calculated at 130K (red curve).

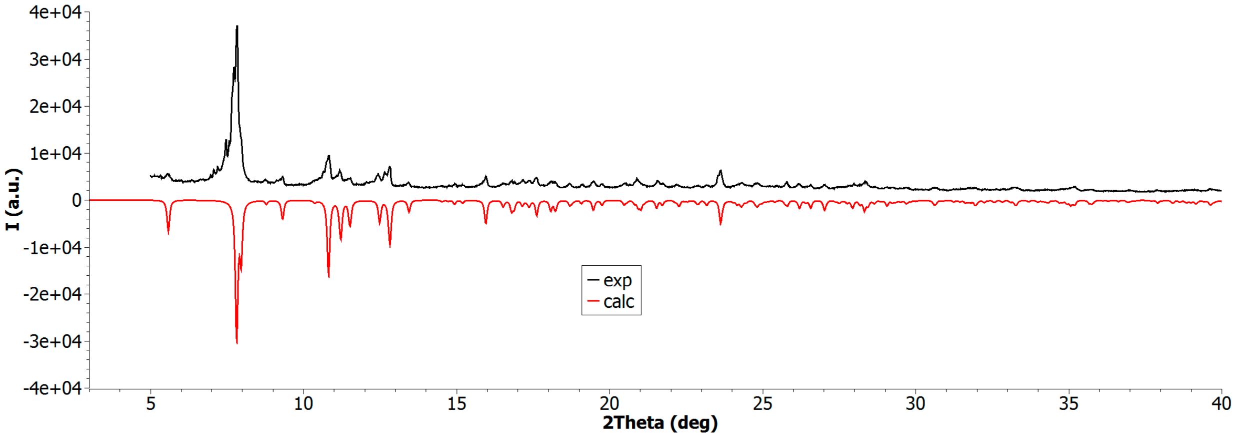

Figure S2. Powder patterns comparison for **4**: experimental at 298K (black curve), calculated at 298K (red curve).

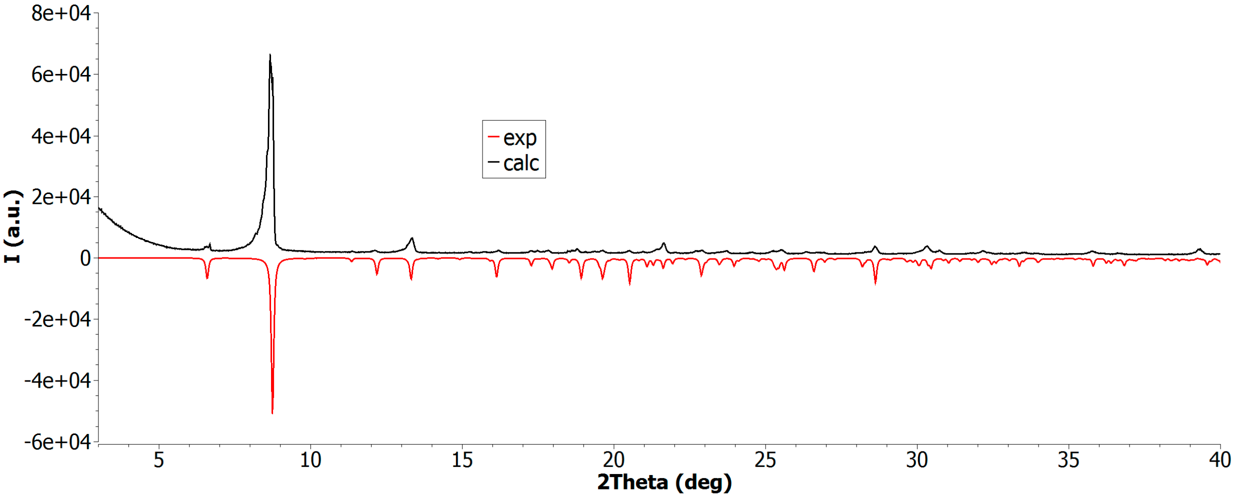

Figure S3. Powder patterns comparison for **5**: experimental at 298K (black curve), calculated at 130K (red curve).

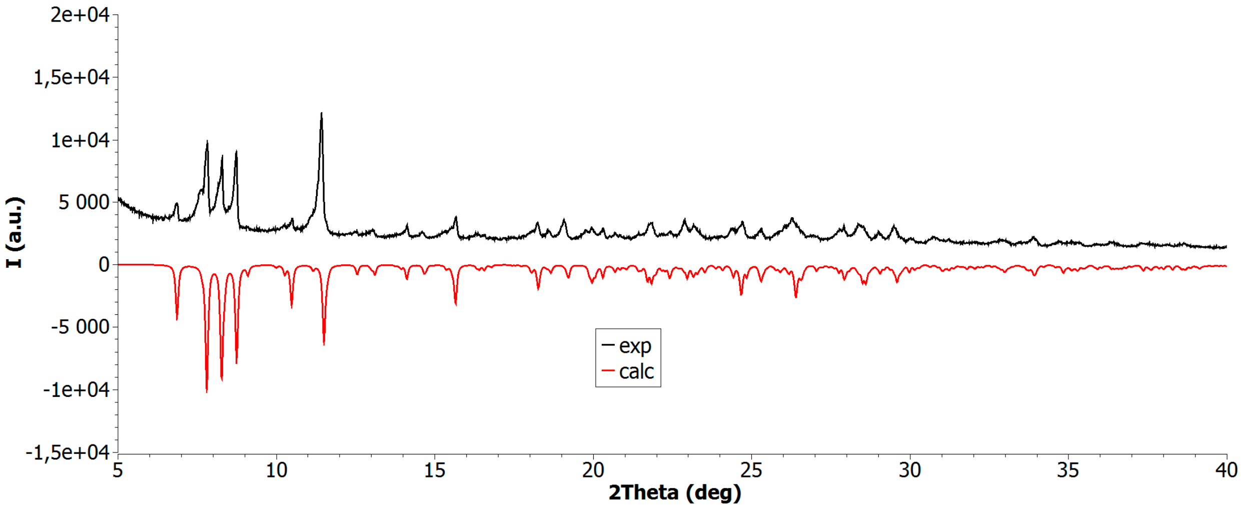

Figure S4. Powder patterns comparison for **6**: experimental at 298K (black curve), calculated at 296K (red curve).

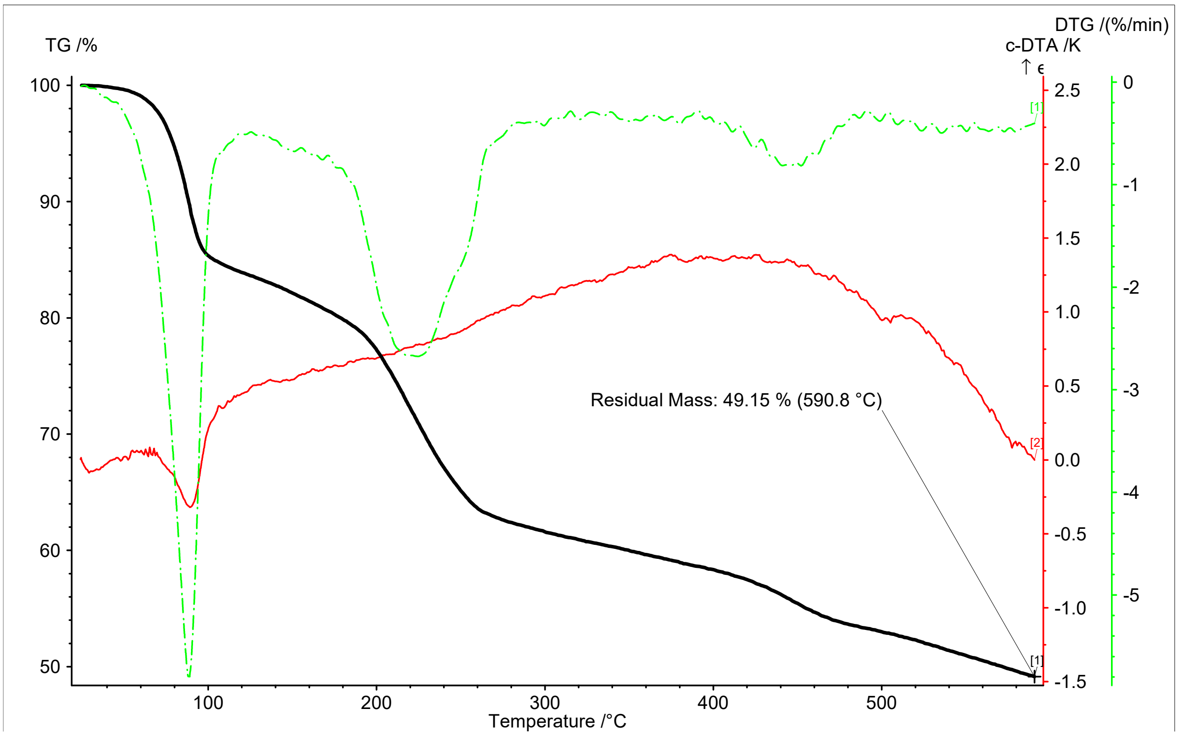

Figure S5. TGA data for **1**.

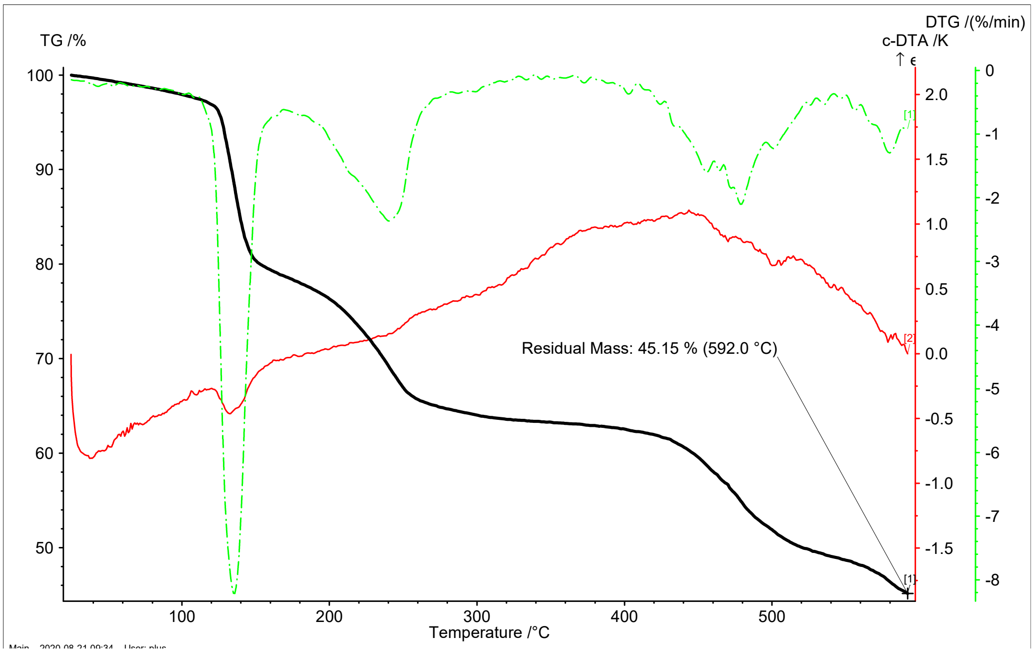

Figure S6. TGA data for **2**.

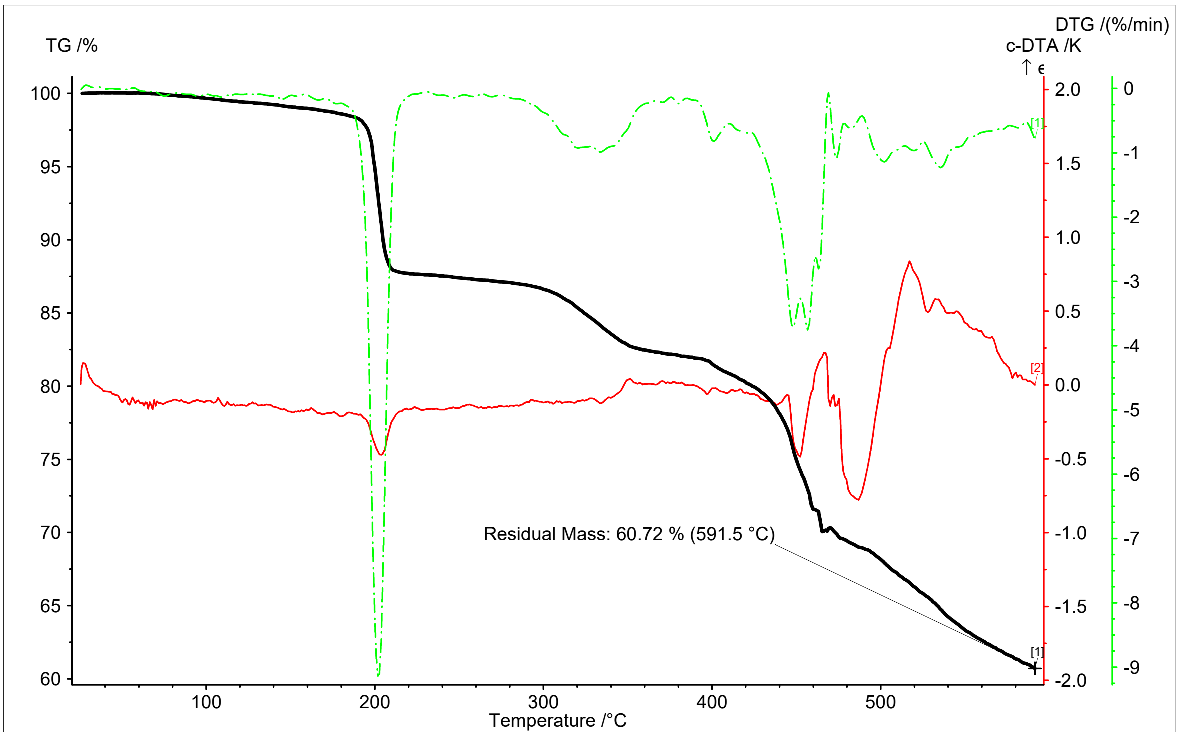

Figure S7. TGA data for **3**.

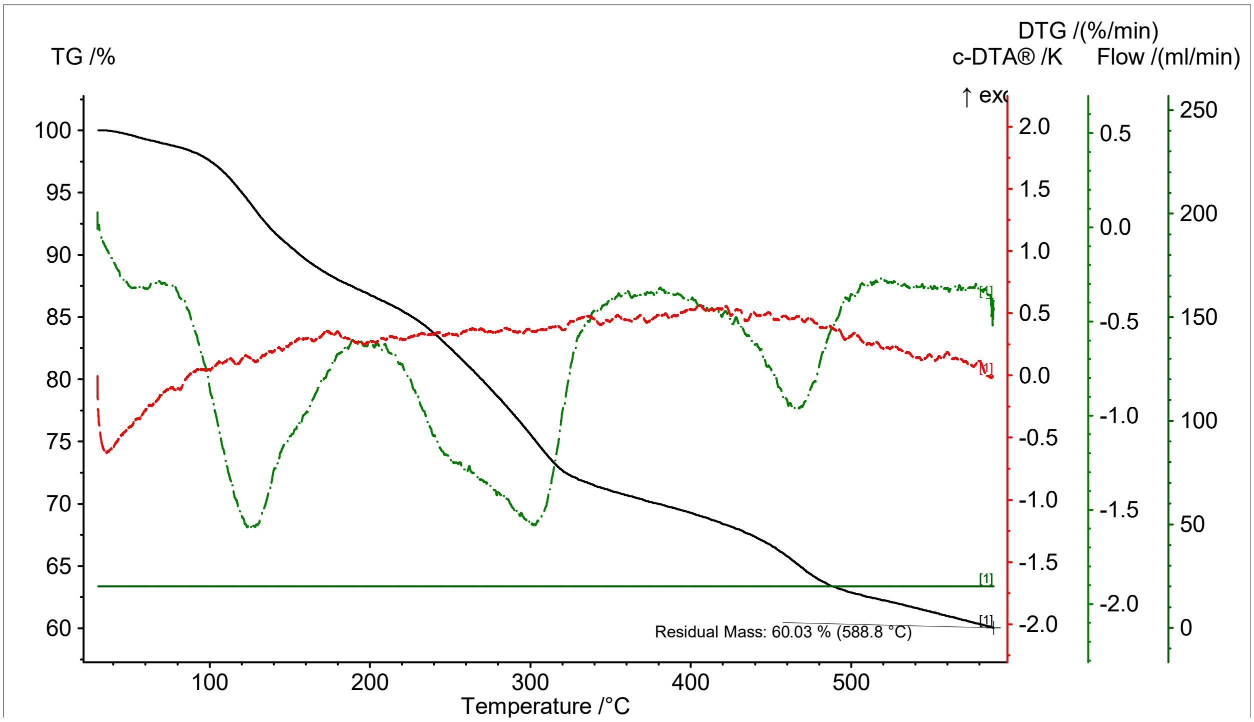

Figure S8. TGA data for **4**.

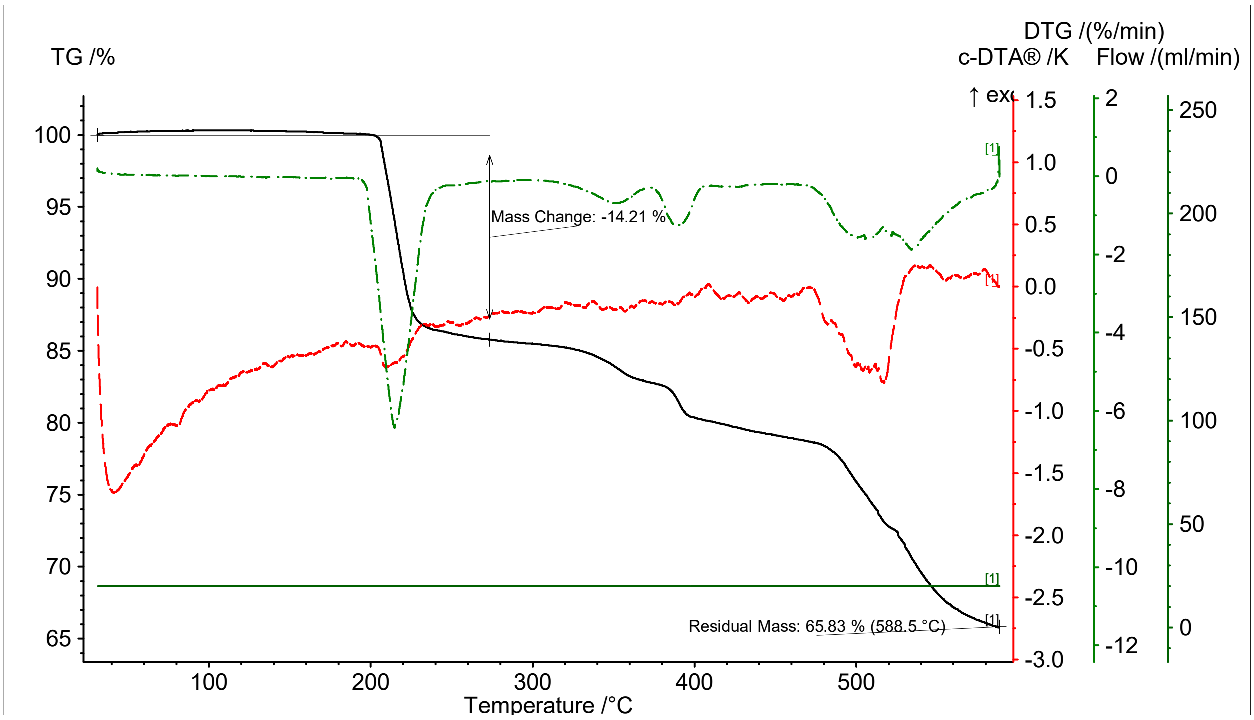

Figure S9. TGA data for **5**.

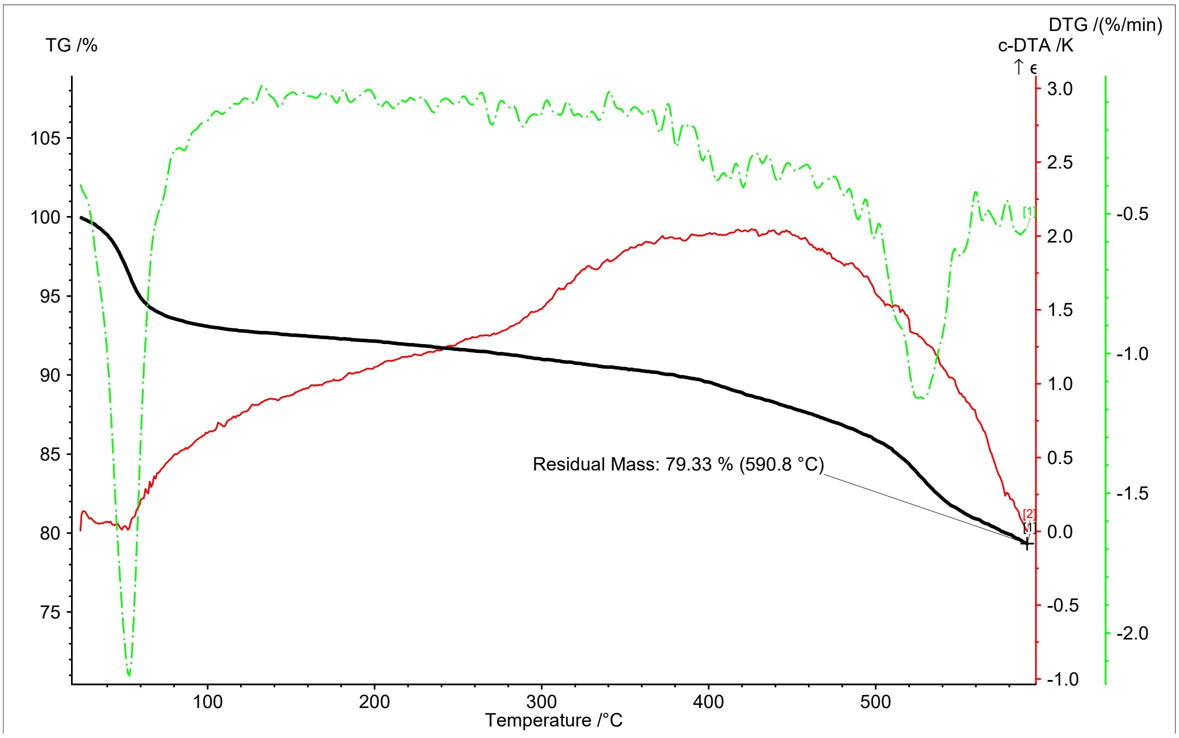

Figure S10. TGA data for **6**.

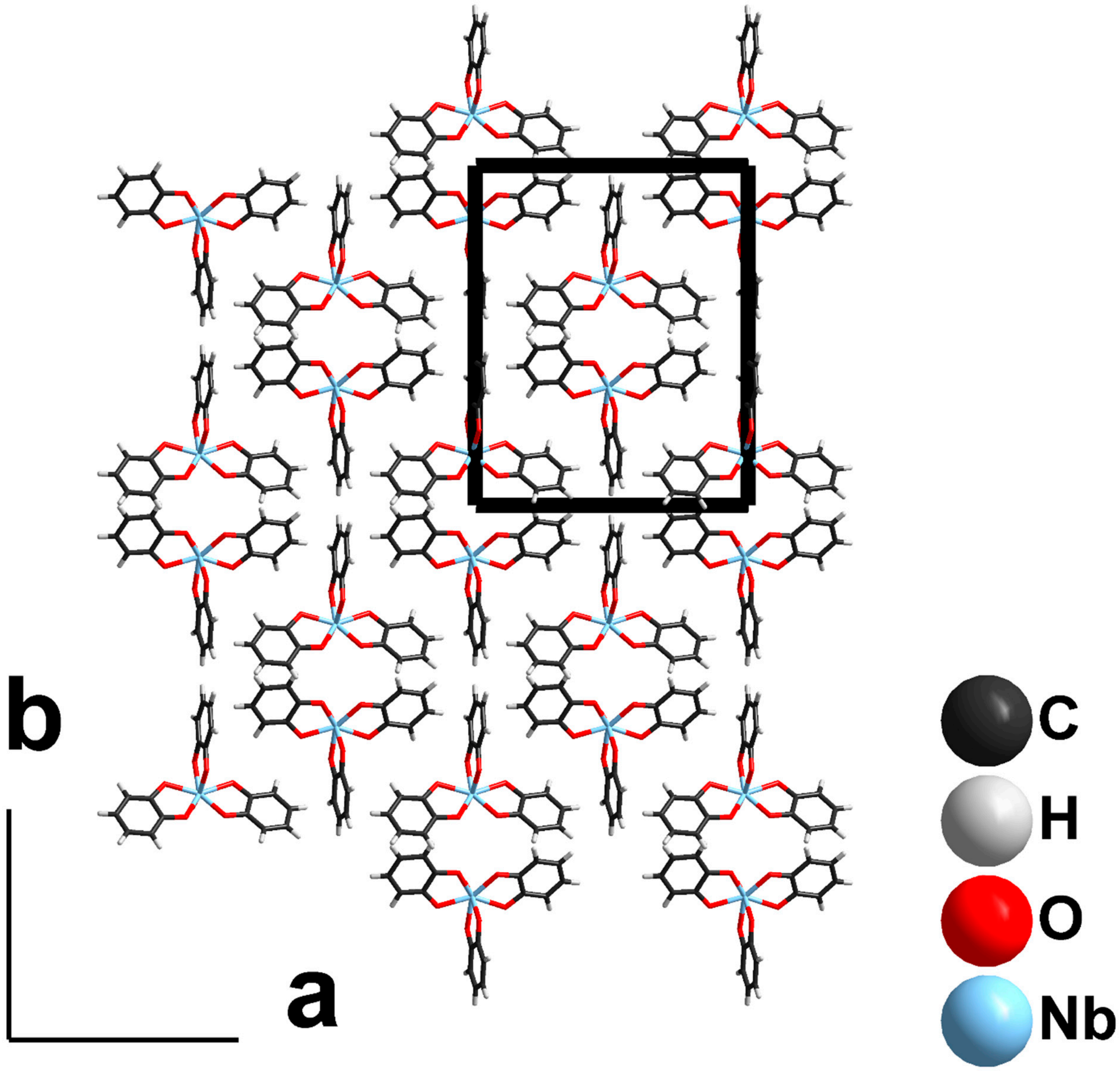

Figure S11. Crystal packing of **1**.

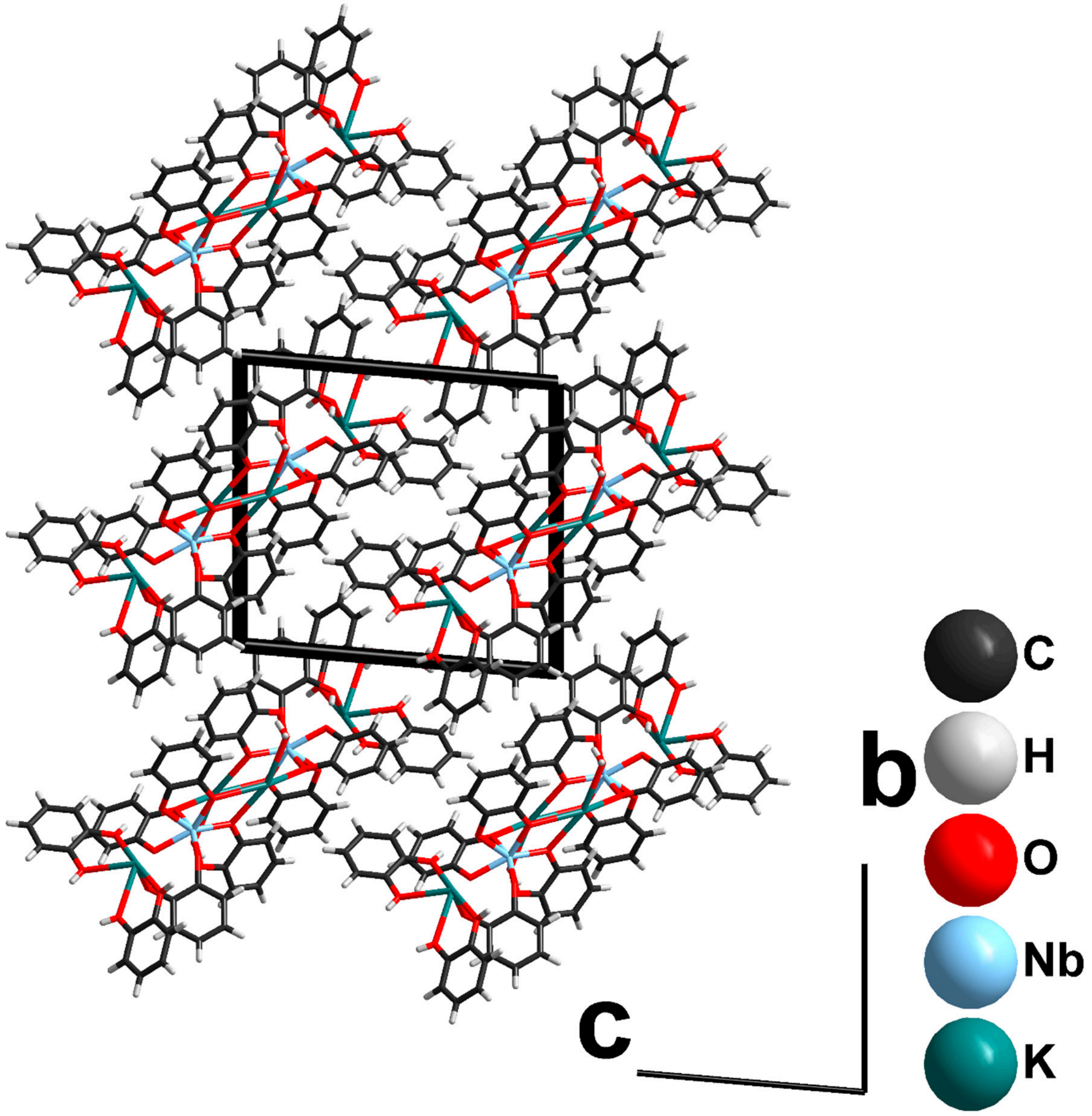

Figure S12. Crystal packing of **2**.

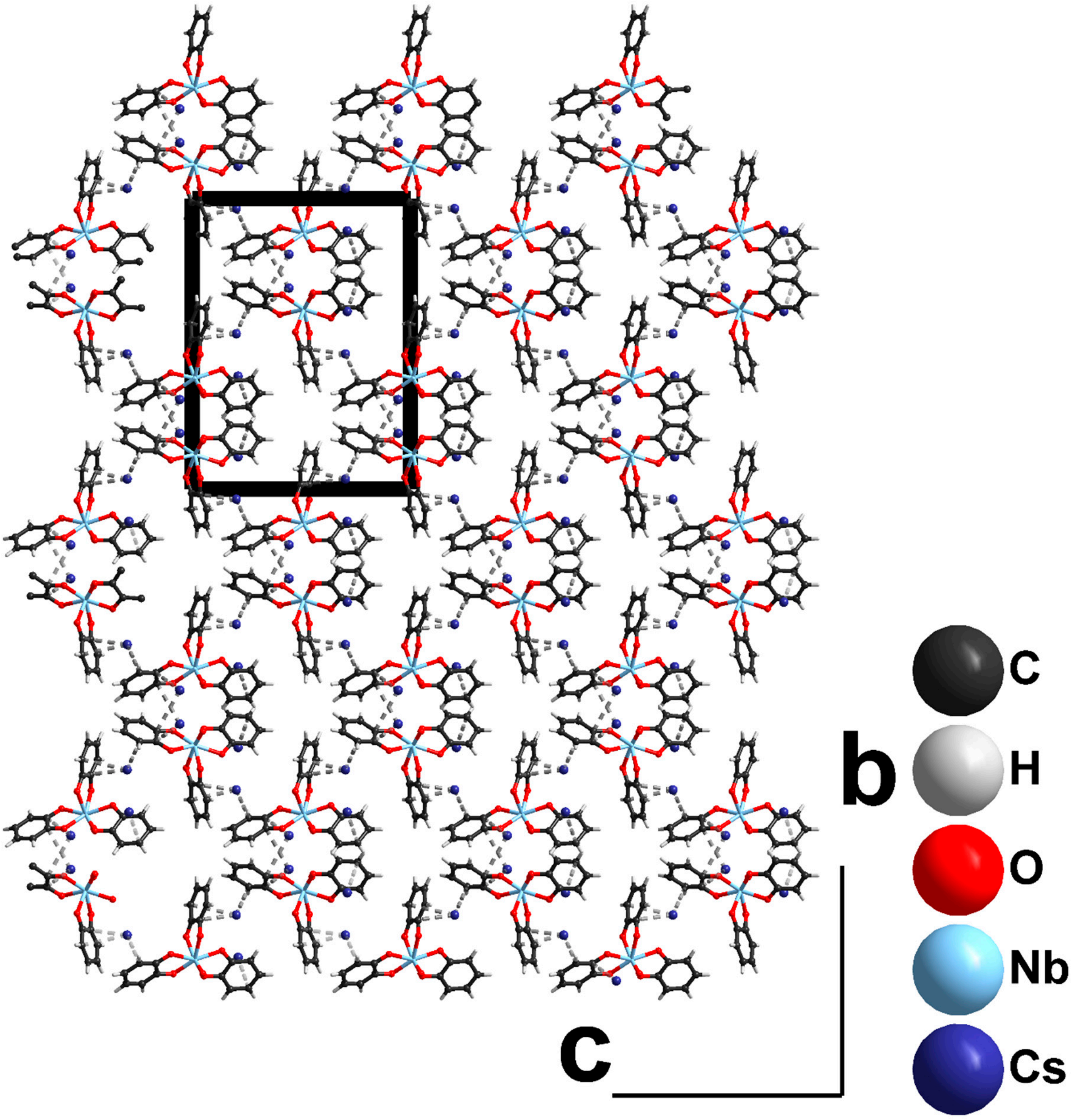

Figure S13. Crystal packing of **3**.

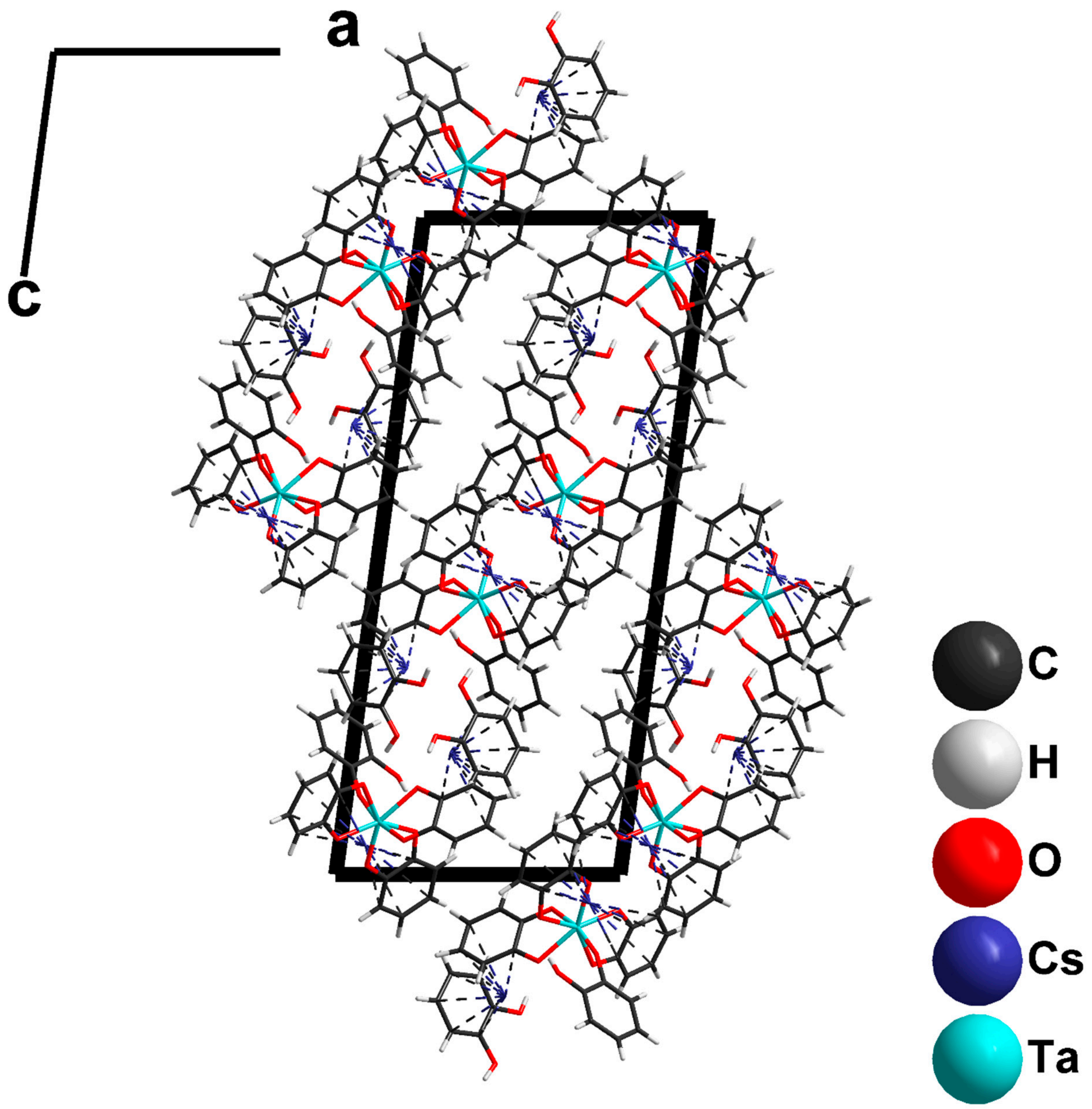

Figure S14. Crystal packing of **5**.

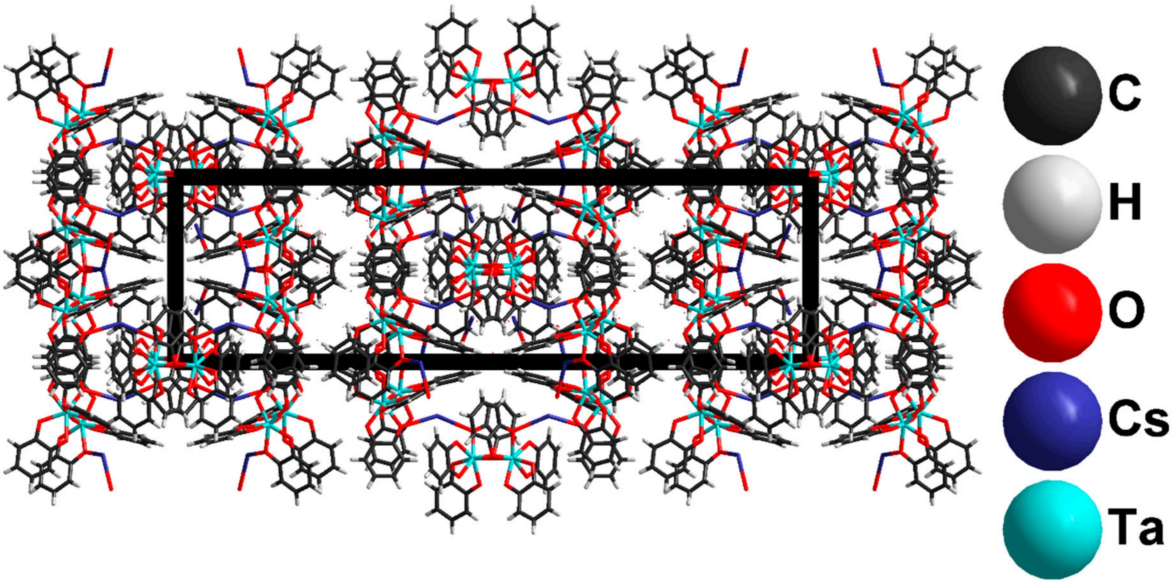

Figure S15. Crystal packing of **6**.

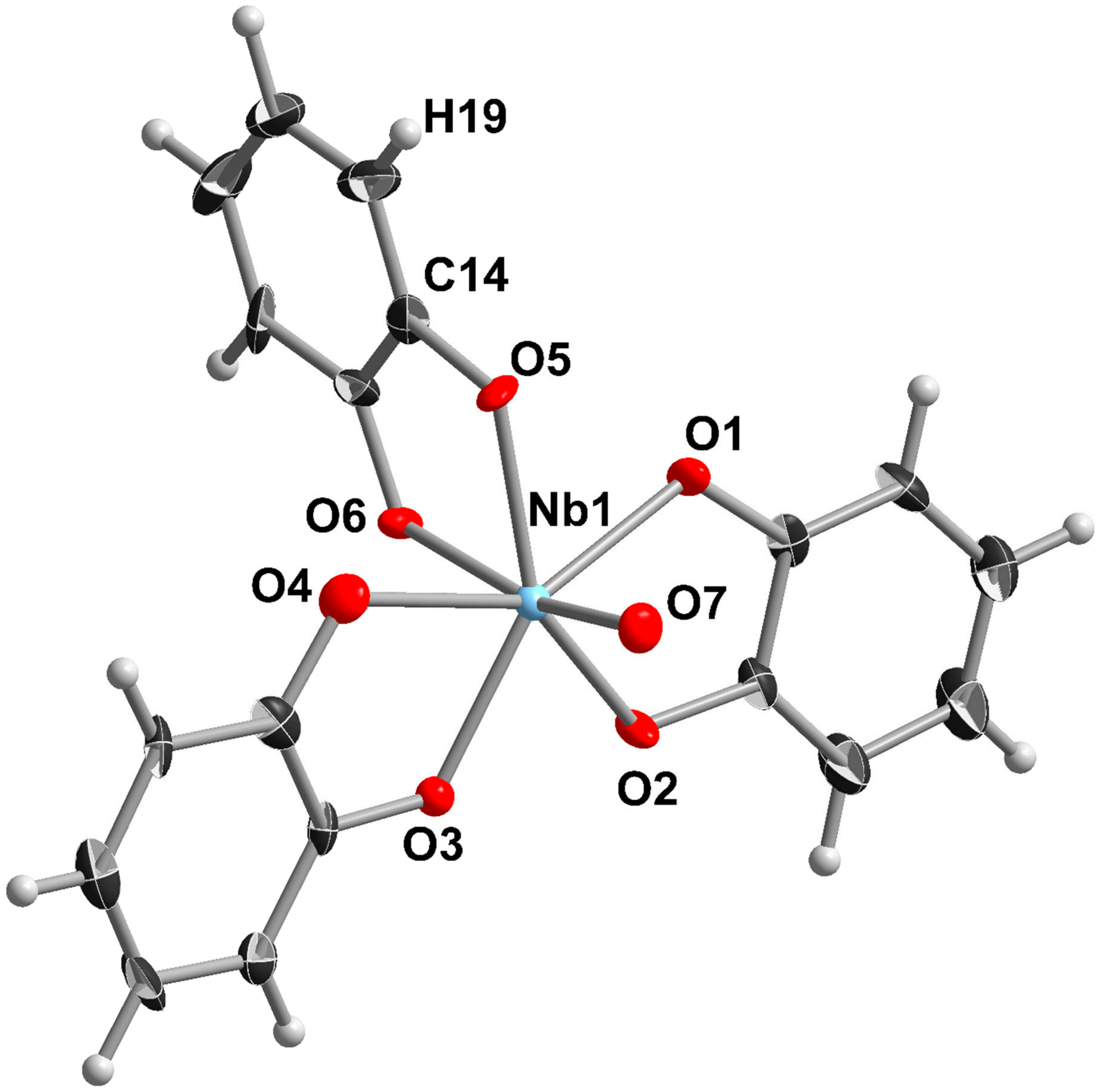

Figure S16. Ellipsoid representation of $[NbO(cat)_3]^{3-}$ in the crystal structure of **1**.

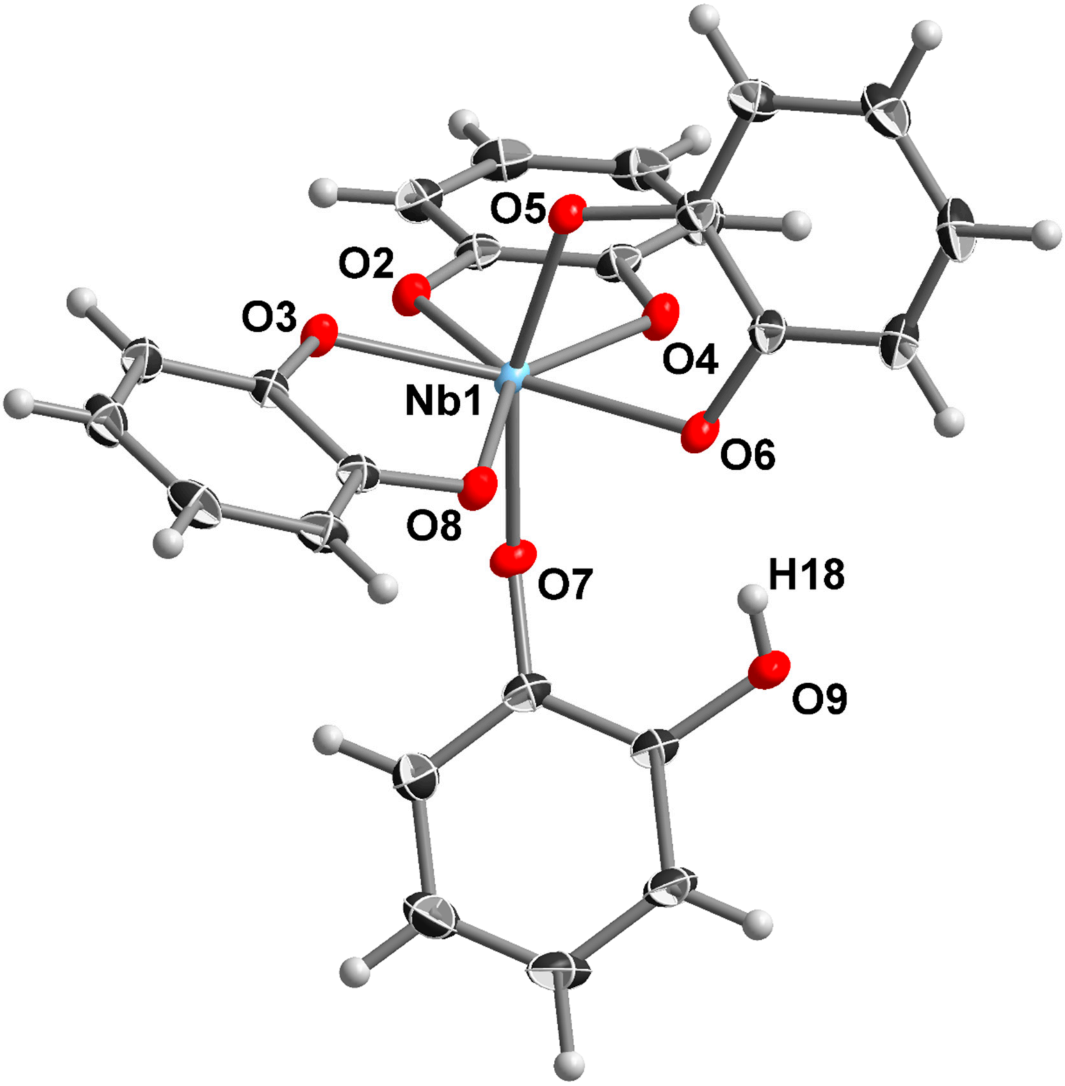

Figure S17. Ellipsoid representation of $[Nb(cat)_3(Hcat)]^{2-}$ in the crystal structure of **2**.

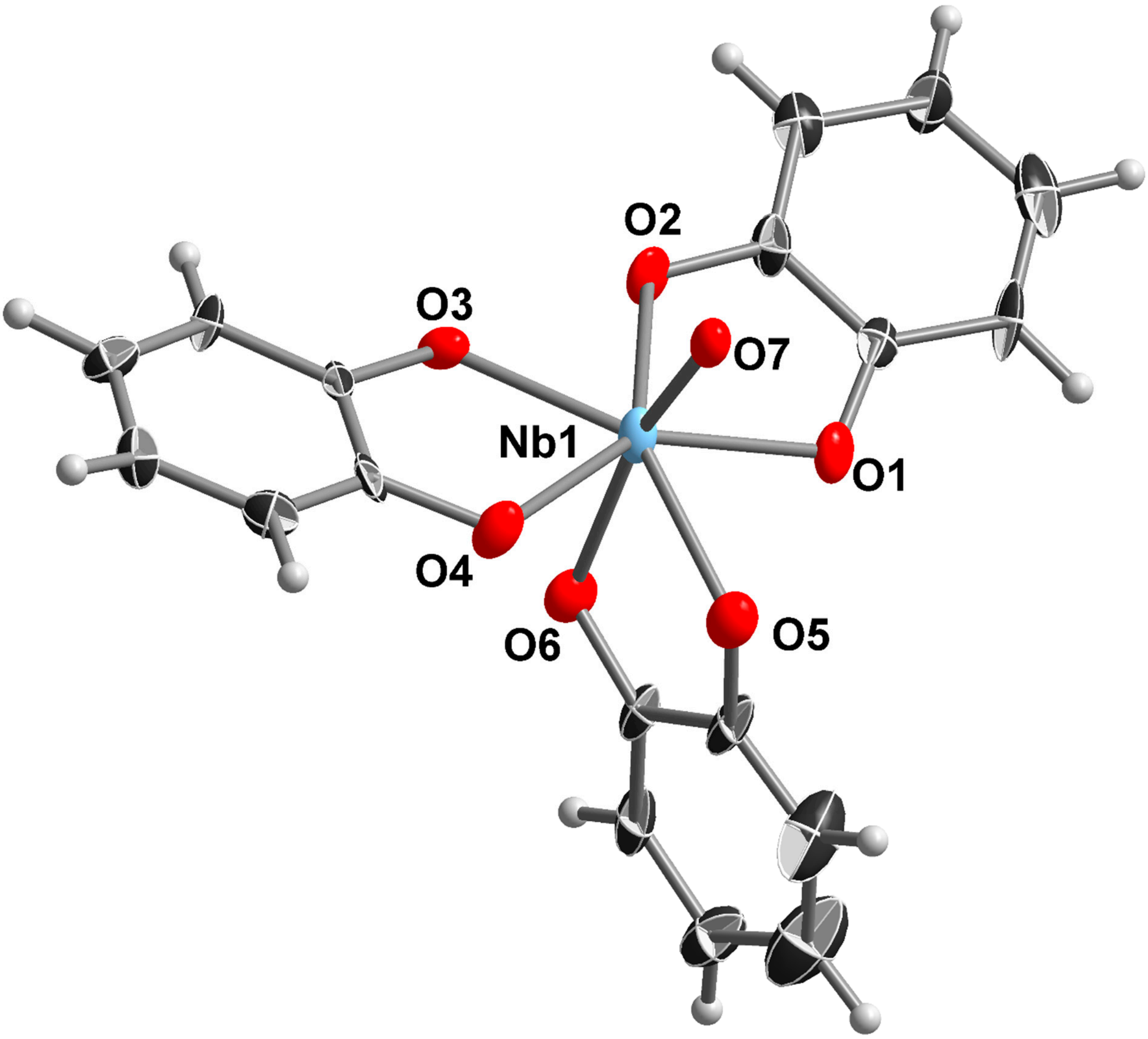

Figure S18. Ellipsoid representation of $[NbO(cat)_3]^{3-}$ in the crystal structure of **3**.

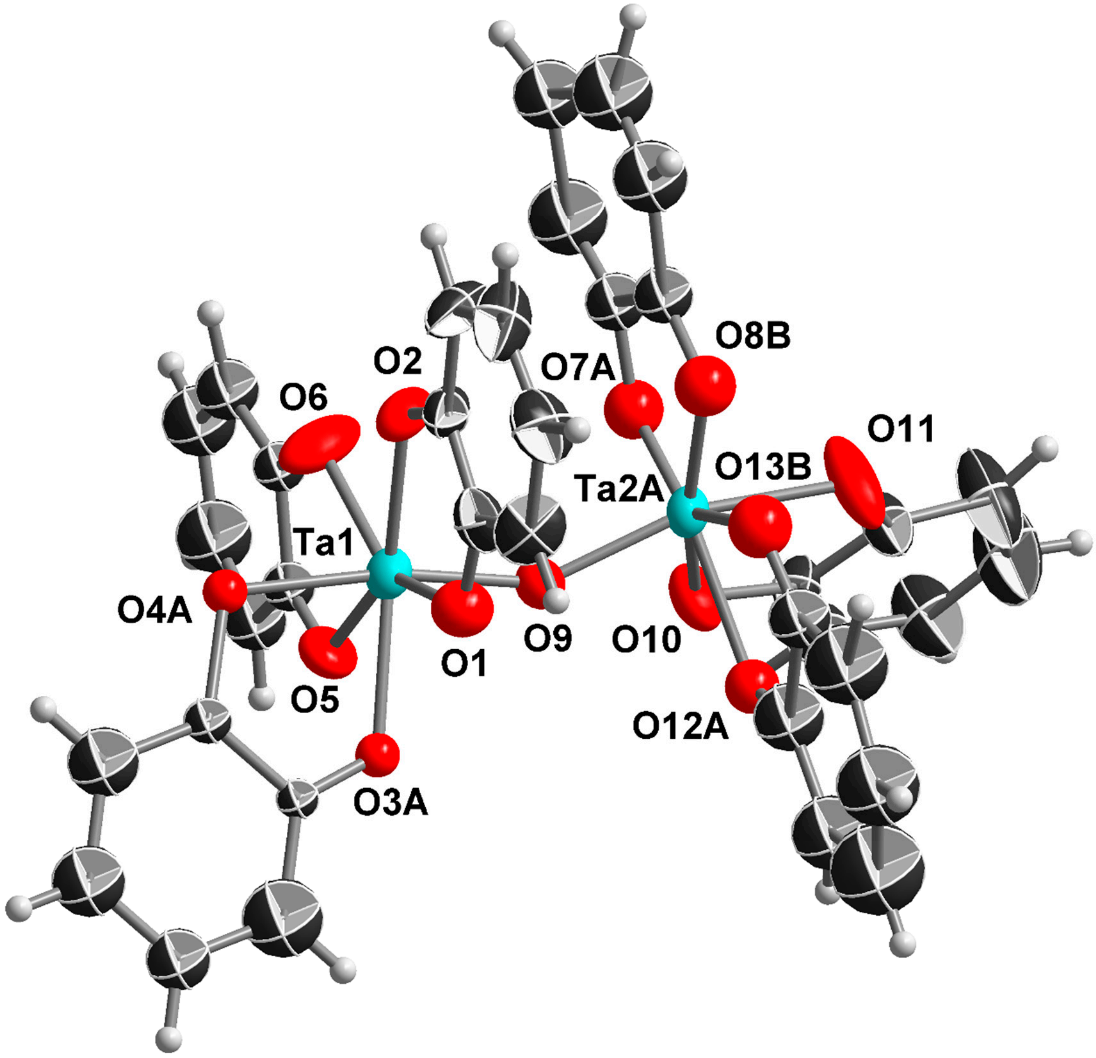

Figure S19. Ellipsoid representation of $[Ta_2O(cat)_6]^{4-}$ in the crystal structure of **4**.

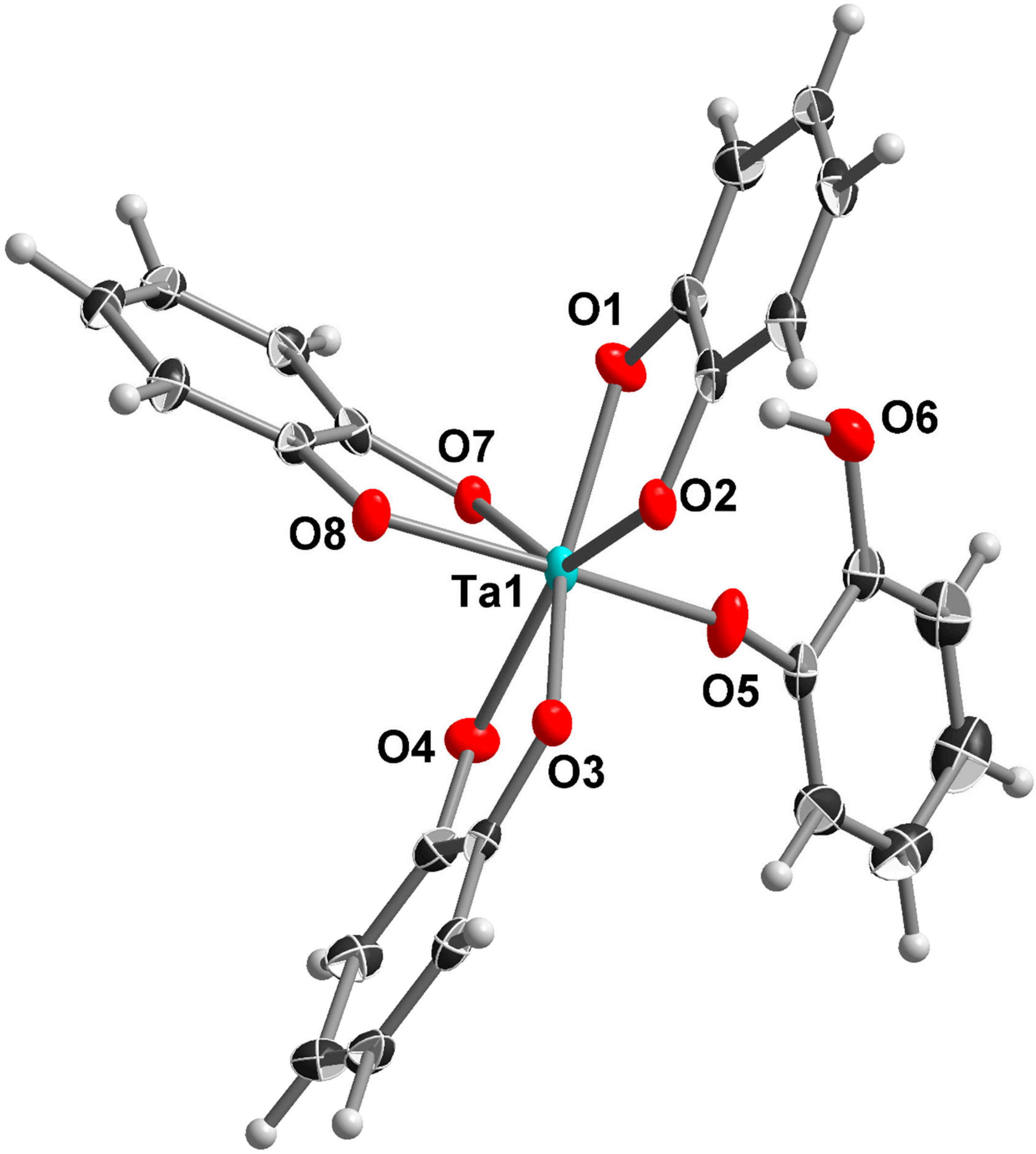

Figure S20. Ellipsoid representation of $[Ta(cat)_3(Hcat)]^{2-}$ in the crystal structure of **5**.

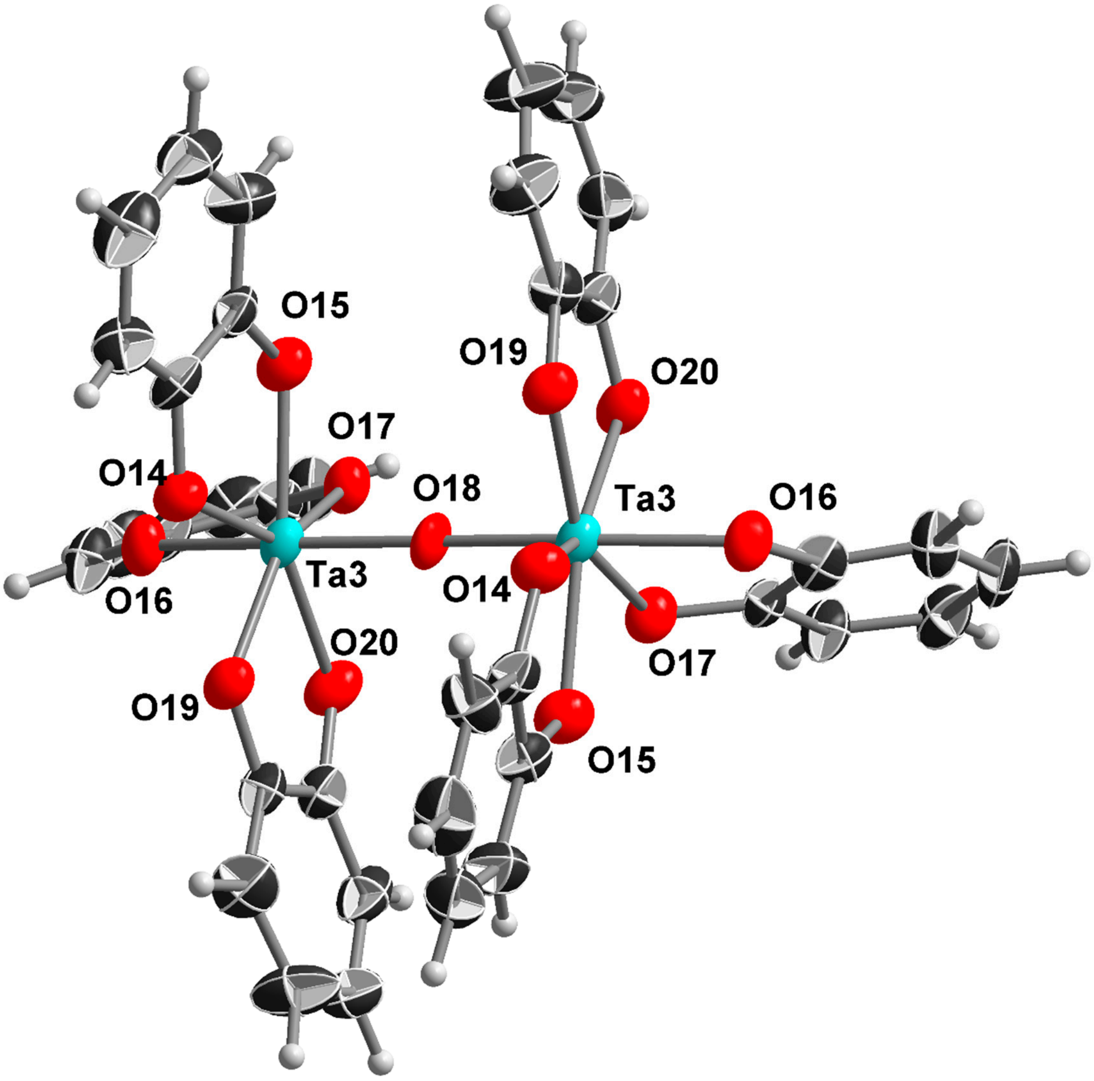

Figure S21. Ellipsoid representation of $[Ta_2O(cat)_6]^{4-}$ in the crystal structure of **6**.
